# Insights into the Mechanisms Behind Structural Repair of Spent Layered Cathode Materials for Lithium‐Ion Batteries

**DOI:** 10.1002/anie.202504382

**Published:** 2025-06-22

**Authors:** Shuaiwei Liu, Hao Liu, Arseniy Bokov, Mohammad Jaleh, Hang Li, Sylvio Indris, Oleksandr Dolotko, Aleksandr Kalinko, Edgar Eduardo Villalobos Portillo, Carlo Marini, Thomas Bergfeldt, Michael Knapp, Helmut Ehrenberg

**Affiliations:** ^1^ Institute for Applied Materials (IAM) Karlsruhe Institute of Technology (KIT) Hermann‐von‐Helmholtz‐Platz 1, D‐76344 Eggenstein‐Leopoldshafen Karlsruhe Germany; ^2^ Helmholtz‐Institute Ulm for Electrochemical Energy Storage (HIU) P.O. Box 3640 D‐76021 Karlsruhe Germany; ^3^ Applied Chemistry and Engineering Research Centre of Excellence (ACER CoE) Université Mohammed VI Polytechnique (UM6P) Lot 660, Hay Moulay Rachid Ben Guerir 43150 Morocco; ^4^ Deutsches Elektronen‐Synchrotron (DESY) Notkestrasse 85 Hamburg Germany; ^5^ CELLS‐ALBA, Synchrotron Barcelona E‐08290 Spain

**Keywords:** Direct recycling, Layer cathodes, Spent lithium‐ion battery, Structural repair

## Abstract

Structural repair is a vital step in the direct recycling of spent LiNi*_x_* Co*_y_* Mn*_z_* O_2_ lithium‐ion batteries, yet its underlying mechanisms remain insufficiently clear. Herein, the thermal solid‐state structural repair of spent LiNi_0.6_Co_0.2_Mn_0.2_O_2_ (NCM622) layered cathode material is systematically investigated. Through multiscale techniques combining XRD, XAS, and ^6^Li solid‐state NMR, we identify the structural degradation in spent NCM622 and monitor both long‐ and short‐range structural evolution during repair. Our findings reveal that degradation predominantly occurs through Ni migration into Li octahedral sites, while Co and Mn demonstrate relatively lower occupancies in the Li layer. Such occupancies are primarily responsible for structural disorder and cubic‐symmetry domain formation within the spent material. The repair process is demonstrated to involve re‐lithiation, oxygen capture, increased transition metal (TM) oxidation states, and the migration of TM ions from the Li layer back to the TM layer, followed by cation diffusion. Both temperature and lithium compensation ratio are identified as critical factors promoting these processes. Capacity recovery studies show a strong correlation between reduced TM occupancy in the Li layer and improved electrochemical performances. These insights allow us to move beyond conventional phase‐transition perspectives, offering an atomic‐level understanding of structural degradation and repair mechanisms in spent layered cathode materials.

## Graphical abstract

Structural repair is key to direct recycling, yet its mechanisms remain unclear. By combining XRD, XAS, and ^6^Li solid‐state NMR, this study identifies a repair process involving re‐lithiation, oxygen capture, transition‐metal oxidation, and migration for LiNi_0.6_Co_0.2_Mn_0.2_O_2_ layered cathode —governed by temperature and lithium compensation. An atomic‐level repair mechanism is thus proposed, offering valuable insights into cathode performance recovery.

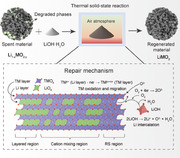

## Introduction

Recycling of spent lithium‐ion batteries (LIBs) is attracting growing attention from both academia and industry, due to environmental concerns and the need for sustainable resource utilization.^[^ ^1^, ^2^ ^]^ However, traditional metallurgy‐based recycling technologies, which dominate current industries,^[^ ^3^, ^4^ ^]^ face challenges related to high energy consumption and waste generation. In this context, direct recycling is gaining attention as a promising alternative,^[^ ^5^ ^]^ and is currently in the early stages of commercialization. This approach offers lower energy consumption and improved economic efficiency by preserving the original structure of cathode active material (CAM) and regenerating it to its pristine functionality through the repair of structural and chemical deficiencies, without decomposing the material into its individual elements.^[^ ^6^, ^7^ ^]^

Recent efforts in direct recycling have primarily focused on the structural and chemical repair process of spent layered LiNi*_x_* Co*_y_* Mn*_z_* O_2_ (NCM, *x* + *y* + *z* = 1),^[^ ^8^, ^9^, ^10^, ^11^ ^]^ likely because of being the dominant CAM employed in electric vehicles. The studies over the past few years indicate that the repair processes primarily involve a local phase transformation from degraded phases (rock‐salt/spinel phases) back to the original phase (layered phase), driven by re‐lithiation.^[^ ^12^ ^]^ It is well known that rhombohedral (*R‐3m*), spinel (*Fd‐3m*), and rock‐salt (*Fm‐3m*) structures all rely on a cubic‐close‐packed oxygen framework, with cations occupying tetrahedral or octahedral sites. Consequently, the core of the repair process is fundamentally governed by the kinetics of cation migration and diffusion. Currently, thermal solid‐state reactions are the most commonly employed method to enhance these kinetic processes and drive the reformation of the layered phase.^[^ ^9^, ^13^, ^14^ ^]^

Understanding how degraded phases transform back to the original structure is essential for designing effective regeneration strategies for such spent layered cathode materials. However, due to the similarity in the cubic‐close‐packed oxygen framework in the degraded and layered phases, as well as the localized formation of degraded phases in spent materials, differentiating between these phases can be challenging. Although transmission electron microscopy (TEM) along with Fast Fourier Transform (FFT) analyses, are commonly used to evaluate the success of structural repair,^[^ ^10^, ^15^, ^16^ ^]^ these methods often fail to fully capture the detailed structural and chemical evolution occurring throughout the repair process. As a result, the specific mechanisms involving cation migration and differences in migration behaviors among the three transition metals (TMs) Ni, Co, and Mn remain largely unexplored. Investigating these aspects could provide crucial insights into optimizing repair conditions and improving the effectiveness of direct recycling for NCM materials.

In this work, the structural repair process of spent LiNi_0.6_Co_0.2_Mn_0.2_O_2_ (NCM622) using thermal solid‐state reaction is investigated, focusing specifically on how lithium compensation ratio and reaction temperature affect the structural repair. To gain a comprehensive understanding of structural and chemical changes throughout the repair process, we employ multiscale characterization techniques, including diffraction, absorption spectroscopy, and nuclear magnetic resonance, to illustrate the structural degradation in spent NCM622 and to reveal both long‐ and short‐range structural evolution upon the repair process. Moreover, we assess the capacity recovery of materials regenerated under different reaction conditions, revealing a correlation between capacity recovery and structural order. Finally, a possible thermal solid‐state reaction mechanism for the structural repair, involving re‐lithiation, oxygen capture, TM oxidation, and migration, is proposed. These findings provide essential insights into structural repair mechanisms and contribute to advancing sustainable battery recycling technologies.

## Results and Discussions

**Figure 1:**
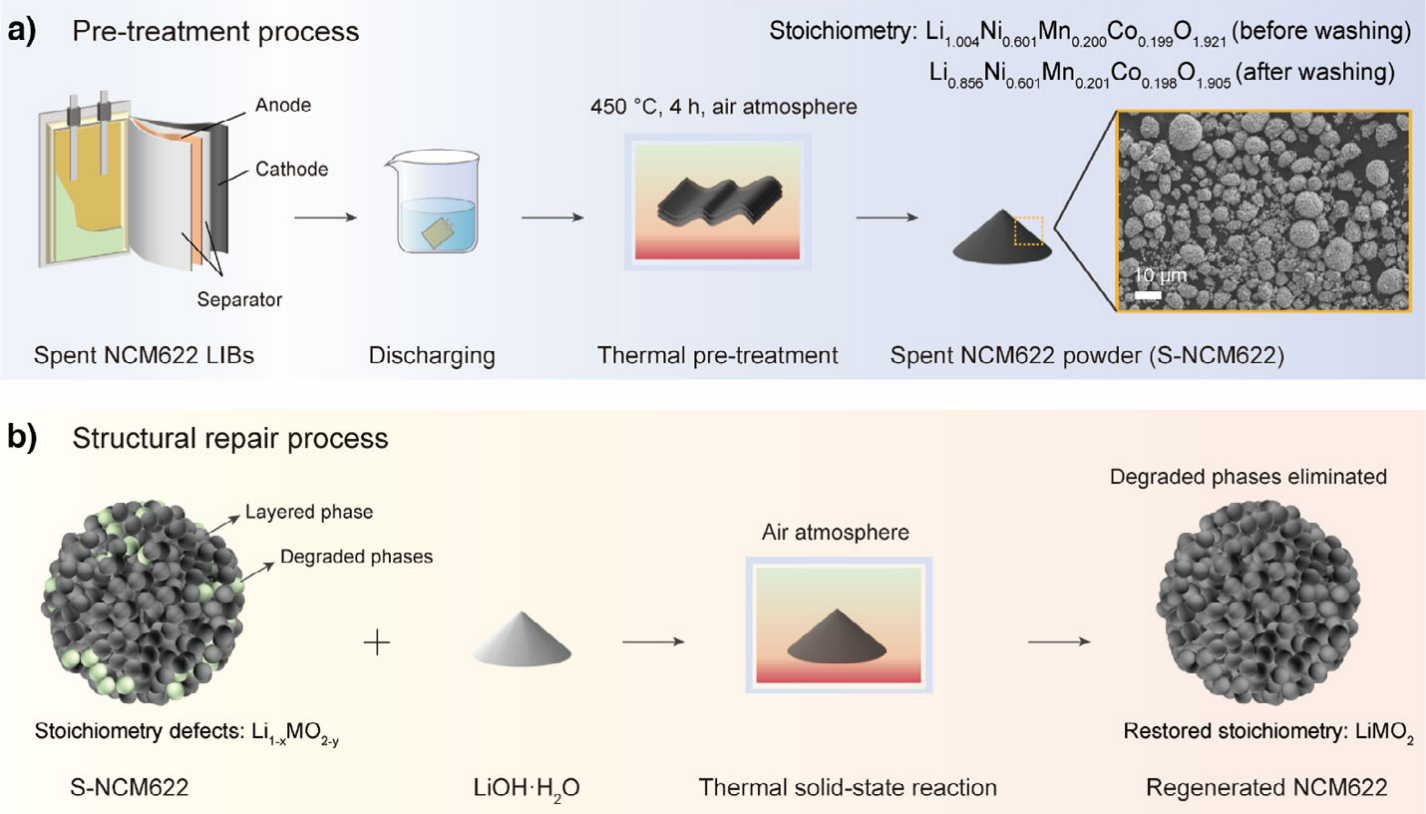
Schematic illustration for a a) pre‐treatment process and b) subsequent structural repair process used in the present work.

In the present work, the spent NCM622 powder (denoted as S‐NCM622) was obtained from an end‐of‐life pouch cell (retired after thousands of cycles with a capacity retention of 81%) via thermal treatment at 450 °C for 4 h in an air atmosphere,^[^ ^17^ ^]^ as shown in Figure 1a. S‐NCM622 exhibits a typical polycrystalline morphology but with obvious particle cracks, most likely ascribed to the long‐term cycling.^[^ ^18^, ^19^ ^]^ Its stoichiometry is confirmed as Li_1.004_Ni_0.601_Mn_0.200_Co_0.199_O_1.921_ (Table S1) through Inductively Coupled Plasma Optical Emission Spectrometry (ICP‐OES) and Carrier Gas Hot Extraction (CGHE). After a water‐washing process to remove the potentially residual lithium salts (e.g., LiOH, Li_2_CO_3_, and Li_3_PO_4_) from the surface, this stoichiometry shifts to Li_0.856_Ni_0.601_Mn_0.201_Co_0.198_O_1.905_ (Table S2)_,_ suggesting lithium and oxygen loss from the structure. It is important to note that the unwashed material is primarily used for analysis and regeneration in this study. Besides, no significant stoichiometry changes are observed for the TM elements.

To better understand the structural and chemical changes in S‐NCM622, pristine NCM622 (denoted as P‐NCM622), commercially purchased, is employed as a reference. Its stoichiometry is found to be Li_1.046_Ni_0.602_Mn_0.200_Co_0.198_O_2.036_ (Table S3), aligning well with the standard stoichiometry of NCM622. Electrochemical studies on P‐NCM622 using a half‐cell configuration reveal a discharge capacity of 170.1 mAh g^−1^ at its initial 0.1 C (1 C = 160 mA g^−1^) cycle over 3.0–4.3 V, and a capacity retention of 96% after 100 cycles at 1 C charge/discharge within the same voltage range, as illustrated in Figure S1a,b.

The structural repair process of S‐NCM622 was realized by a thermal solid‐sate reaction, as depicted in Figure 1b. Specifically, S‐NCM622 was homogeneously mixed with LiOH·H_2_O, and then was heated under an air atmosphere in a muffle furnace to restore its structure and stoichiometry.

### Structural Degradation in Spent NCM622 Cathode Material

**Figure 2:**
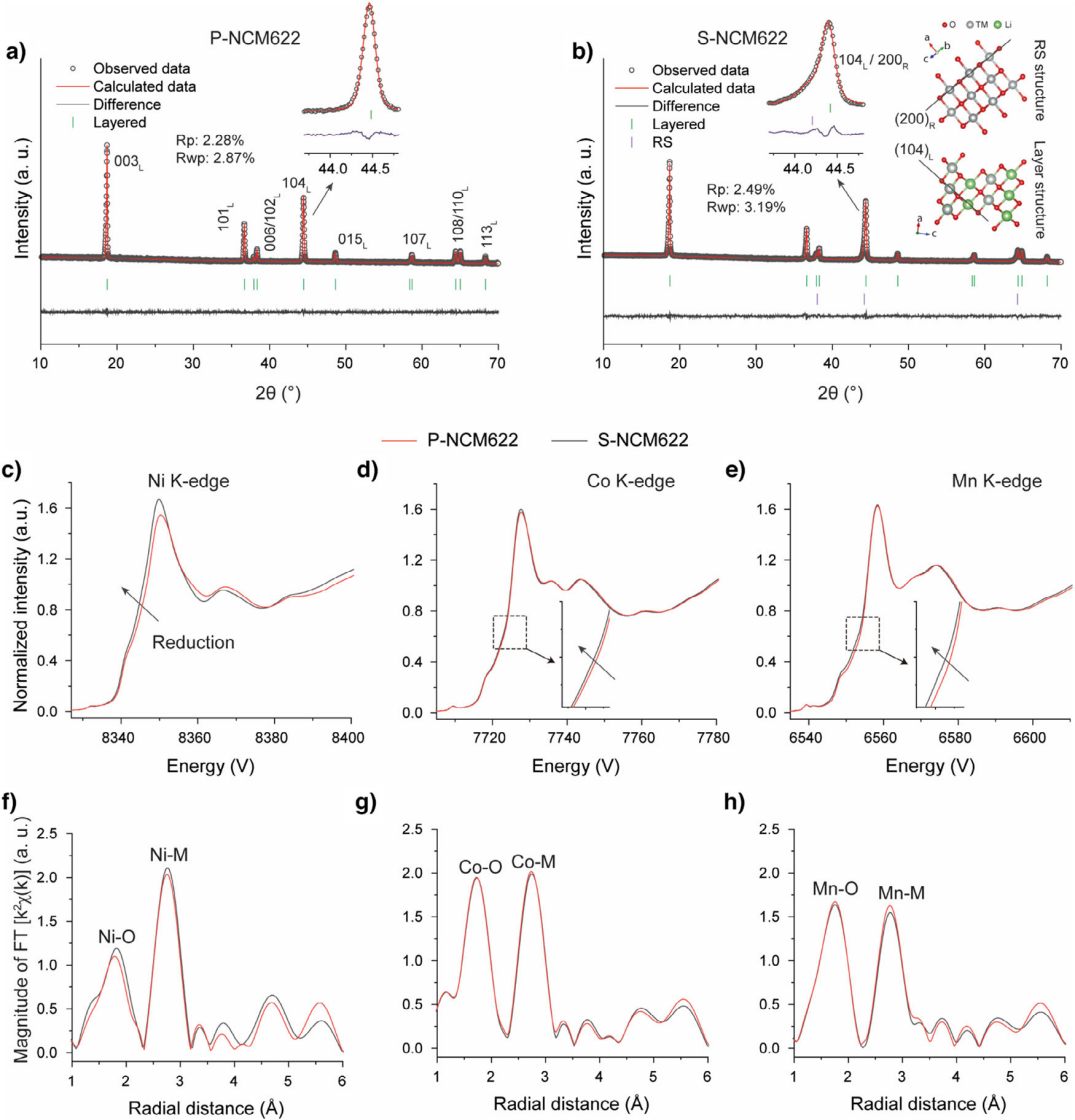
XRD patterns (Cu‐Kα_1_ radiation, λ = 1.54056 Å) and corresponding Rietveld refinement results for a) pristine NCM622 (P‐NCM622) and b) spent NCM622 (S‐NCM622). Normalized XANES spectra of c) Ni, d) Co, and e) Mn K‐edge for P‐NCM622 and S‐NCM622. EXAFS results of f) Ni, g) Co, and h) Mn K‐edge for P‐NCM622 and S‐NCM622.

To clarify the structural repair process, it is crucial to first understand the structural degradation occurring in S‐NCM622. To investigate the structural degradation, X‐ray Diffraction (XRD) analysis was initially conducted on both P‐NCM622 and S‐NCM622. The resulting patterns and Rietveld refinement are presented in Figure 2a,b. As displayed in Figure 2a, P‐NCM622 exhibits the characteristic rhombohedral structure (*R‐3m*) with well‐defined reflections, indicating a highly ordered layered structure. In S‐NCM622, the observed reflections suggest that it retains the layered structure. Nonetheless, the application of a one‐phase (layered phase, *R‐3m*) Rietveld refinement results in a suboptimal fit to the observed peaks. This poor fit can be attributed to profile anomalies in certain reflections (Figure S2a,b), particularly at the 104 _L_ reflection, which represents local destruction of the crystal's rhombohedral symmetry. Importantly, these anomalies are primarily located at positions corresponding to reflections associated with rock‐salt structure (RS, *Fm‐3m*) (Figure S3). This suggests that the profile anomalies most likely arise from contributions by domains exhibiting cubic symmetry in the spent material. To further illustrate this, a two‐phase refinement was then performed (layered and RS). The inclusion of RS improves the fit to the observed peaks, as evidenced by changes in the R_p_ and R_wp_ values from their initial values of 2.88% and 4.34%, to improved values of 2.49% and 3.19%, respectively (Figures 2b and S2b). This indicates that the profile anomalies can be ascribed to overlapping contributions from both phases (layered and RS) with varying degrees of cation ordering. That is to say, RS‐type domains resulting from cation disorder are formed in S‐NCM622. This conclusion is supported by Synchrotron Radiation Diffraction (SRD) results as well (Figure S4).

Generally, the RS structure can be characterized by cations occupying edge‐sharing octahedra in a cubic‐close‐packed (CCP) oxygen framework (Figure S5a).^[^ ^20^, ^21^, ^22^ ^]^ Although layered NCM materials with rhombohedral symmetry, as shown in Figure S5b, feature alternating Li and TM ions occupying edge‐sharing octahedra along (104)_L_ plane, also in an CCP oxygen framework, but with slight distortion that locally disrupts the CCP symmetry (due to the Jahn–Teller effect of Ni^3+^: 3d^7^, particularly pronounced in high‐nickel materials).^[^ ^23^, ^24^ ^]^ As such, the formation of RS–type domains in S‐NCM622 can be fundamentally understood as TM migration and occupying the Li octahedral site (as schematically shown in Figure S6), coupled with Li and O loss (as evidenced by ICP‐OES and CGHE results), possibly along with an intensified or alleviated CCP oxygen framework distortion in NCM622.

Moreover, such migration is typically accompanied by a reduction in TM oxidation states.^[^ ^25^, ^26^ ^]^ Based on this characteristic, chemical valence study using Hard X‐ray Absorption Spectroscopy (XAS) with focus on X‐ray Absorption Near Edge Structure (XANES) was conducted, to further understand the formation of RS‐type domains in S‐NCM622 and the potential migration differences among Ni, Co, and Mn. The XANES results for both P‐NCM622 and S‐NCM622 in Figure S7 were sequentially collected alongside SRD (as shown in Figure S4). The corresponding Ni, Co, and Mn K‐edge XANES spectra reveal that S‐NCM622 exhibits edge shifts toward lower energy across all three edges compared to P‐NCM622, suggesting decreased average oxidation states of TMs in S‐NCM622.^[^ ^27^, ^28^ ^]^ Notably, the Ni K‐edge exhibits more significant edge shifts than that in Co and Mn K‐edge, most likely indicating that the formation of RS‐type domains in S‐NCM622 resulting from TM migration is largely induced by Ni.

The XANES results for P‐NCM622 and S‐NMC622 measured at PETRA III P64 indicate similar conclusions (Figure 2c–e). To obtain atomic‐level insights into the coordination environment changes of Ni, Co, and Mn during RS‐type domain formation, we analyzed the corresponding Extended X‐ray Absorption Fine Structure (EXAFS, Fourier transformation *k^2^* |*χ(k)*|). Both samples display two dominant peaks in the radial distribution function for each metal edge at ∼1.8 and ∼2.8 Å, assigned to metal–oxygen (M–O) and metal‐metal (M─M) bonds, respectively.^[^ ^29^, ^30^ ^]^ As observed, the Ni─O peak of S‐NCM622 displays a substantial magnitude increase relative to P‐NCM622, while the Co/Mn─O peak shows only minor differences. These findings suggest that RS‐type domain formation involves changes in NiO_6_ octahedral coordination environment, while leaving the Co and Mn local structures relatively unaffected. We infer that the structural changes of NiO_6_ octahedra could be related to the transition of Ni^3+^‐O_6_ to a more symmetric Ni^2+^‐O_6_ (Jahn–Teller free),^[^ ^29^, ^31^ ^]^ with subsequent local accumulation of Ni^2+^ in specific regions by cation diffusion along the *a*/*b*‐axis. This accumulation is most likely responsible for the emergence of cubic‐symmetry domains, while simultaneously inducing locally geometric symmetry changes in the CCP oxygen framework of NCM622 material. In contrast, Co and Mn are believed to exhibit slight variations in their local environments only, largely due to their limited migration and the inherent stability of Co^3+^‐O_6_ and Mn^4+^‐O_6_ octahedra within the TM layer.^[^ ^32^ ^]^ In other words, these observations further suggest that the Ni occupying the Li octahedral site, accompanied by local geometric changes in Ni and O coordination environment, serves as the dominant mechanism driving the RS‐type domain formation in S‐NCM622.

In addition, the increased magnitude observed at ∼3.8 Å from the Ni‐edge spectra for S‐NCM622—a peak generally associated with 180° Ni─O─Ni correlations (Figure S8)—provides further evidence for Ni occupying Li octahedral sites, reinforcing the proposed Ni migration mechanism.^[^ ^33^, ^34^, ^35^ ^]^ Two more distant peaks observed at ∼4.7 and ∼5.5 Å, correspond to combined single M–M and multiple M–M–M scattering between or within TM planes,^[^ ^31^, ^33^ ^]^ offering insights into longer‐range structural changes. These peaks, show similar trends in S‐NCM622 compared to P‐NCM622 across all radial distributions for Ni, Co, and Mn elements, as seen in the corresponding absorption edge spectra. The magnitude variations for S‐NCM622 in these two peaks may be attributed to enhanced interlayer backscattering induced by TM ions in the Li layer (∼4.7 Å), and reduced intralayer backscattering due to Li ions when TM absorbers are located in the Li layer (∼5.5 Å), respectively. As a result, these changes most likely further indicate an overall increase in the amounts of TM ions within the Li layer in S‐NCM622.^[^ ^35^ ^]^

Overall, the migration of TM ions, particularly Ni, from the TM layer to the Li layer, coupled with lithium and oxygen loss, reduction in TM oxidation states, results in local destruction of crystal's rhombohedral symmetry and the formation of RS‐type domains. This is identified as the primary driving force for structural degradation in S‐NCM622. Based on previous studies, the RS‐type domains are believed to be near the material's surface to achieve a low‐energy state.^[^ ^30^, ^36^, ^37^ ^]^ In the following sections, we will explore how this structural degradation can be repaired. The long‐ and short‐range structural evolution during this repair process is examined, and the impact of structural reordering on the recovery of battery capacity of the repaired cathode is discussed.

### Electrochemical Performance Evaluation

To illustrate the mechanisms behind the structural repair process, the restoration of electrochemical performance was first evaluated by measuring the initial discharge capacities at a charge‐discharge rate of 0.1 C (1 C = 160 mA g^−1^) over a voltage range of 3.0–4.3 V. This was done for materials regenerated at various temperatures (100–900 °C, in 50 °C intervals) and with different lithium compensation/TM molar ratios (denoted as Li/TM = 0.1, 0.2 and 0.3, using LiOH·H_2_O as the lithium source) in a half‐cell configuration.

**Figure 3:**
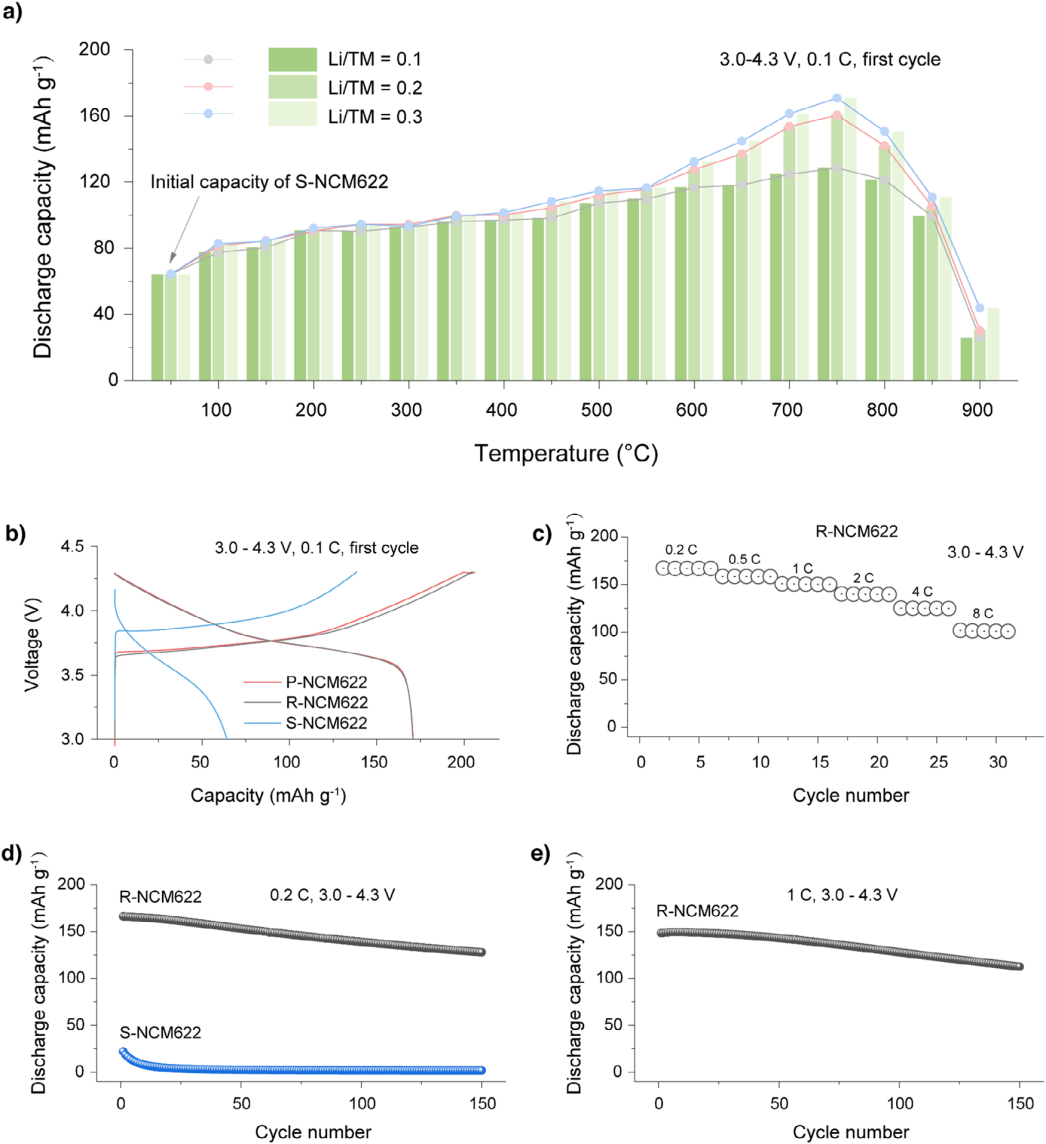
a) Initial discharge capacities at 0.1 C over 3.0–4.3 V for materials regenerated at various temperatures and Li/TM molar ratios (Li/TM refers to lithium compensation/TM molar ratio). b) Initial charge/discharge capacities at 0.1 C over 3.0–4.3 V for S‐NCM622, P‐NCM622, and R‐NCM622 (regenerated under 750 °C with a Li/TM ratio of 0.3). c) Rate the performance of R‐NCM622. d) Cycling performance of R‐NCM622 and S‐NCM622 at charge‐discharge rate of 0.2 C over 3.0–4.3 V. e) Cycling performance of R‐NCM622 and S‐NCM622 at charge‐discharge rate of 1 C over 3.0–4.3 V.

As seen in Figure 3a, S‐NCM622 delivers a discharge capacity of 64.2 mAh g^−1^ in its first cycle at 0.1 C. This low capacity is likely attributed to impurities and further structural degradation introduced during the pretreatment process, in addition to the degradation that occurred during cycling in the initial full‐cell battery. After undergoing the structural repair process, the materials exhibit different levels of capacity recovery. It should be noted that the capacity increases observed below 200 °C is believed to stem from the removal of certain impurities during the washing process following regeneration (Figure S9). The majority of the capacity recovery occurs when the materials are treated between 600 and 750 °C, with higher Li/TM ratios further enhancing the recovery. However, after 750 °C, the capacity recovery begins to decline. Among the regenerated samples, the material regenerated at 750 °C and Li/TM ratio of 0.3 (denoted as R‐NCM622) shows the highest capacity, achieving 170.8 mAh g^−1^, comparable to that of P‐NCM622 (Figure 3b **)**. Further investigation into higher Li/TM ratios (0.4, 0.5, and 0.6) at 750 °C reveals that increasing the Li/M ratio beyond 0.3 does not further improve the capacity (Figure S10). These findings suggest that the optimal condition for achieving maximum capacity recovery in this structural repair process should be at 750 °C with a Li/TM ratio of 0.3.

To further evaluate the restoration of electrochemical properties, the rate and cycling abilities of R‐NCM622 were studied. Figure 3c shows that R‐NCM622 delivers initial discharge capacities of 167.2, 158.4, 150.6, 140.0, 125.3, and 102.0 mAh g⁻¹ at 0.2, 0.5, 1, 2, 4, and 8 C with a cut‐off voltage of 4.3 V, respectively. Figure 3d,e exhibit that R‐NCM622 retains 77% of its initial capacity after 150 cycles at a charge/discharge rate of 0.2 C within the voltage range of 3.0–4.3 V, and 76% capacity retention after 150 cycles at 1 C within the same voltage range. These results further demonstrate the successful restoration of electrochemical properties.

### Analysis of the Restoration Mechanism

With the general understanding of the capacity recovery trend in the regenerated materials, we then carried out a series of in‐depth characterizations to uncover the underlying mechanisms behind the capacity recovery.

Structurally, the repair process can be understood as the reverse of structural degradation. This process generally involves re‐lithiation, oxygen capture, an increase in TM oxidation states, and the TM migration from the Li layer back to the TM layer. This can be represented by the following reaction, where LiOH serves as the lithium source:

$$\begin{array}{ccc} & & {Li}_{1-x}Ni_{0.6}Co_{0.2}Mn_{0.2}O_{2-y}+\mathrm{LiOH}+O_{2}\\ & & ↑\to \mathrm{LiN}i_{0.6}Co_{0.2}Mn_{0.2}O_{2}+H_{2}O↑ \end{array}$$

**Figure 4:**
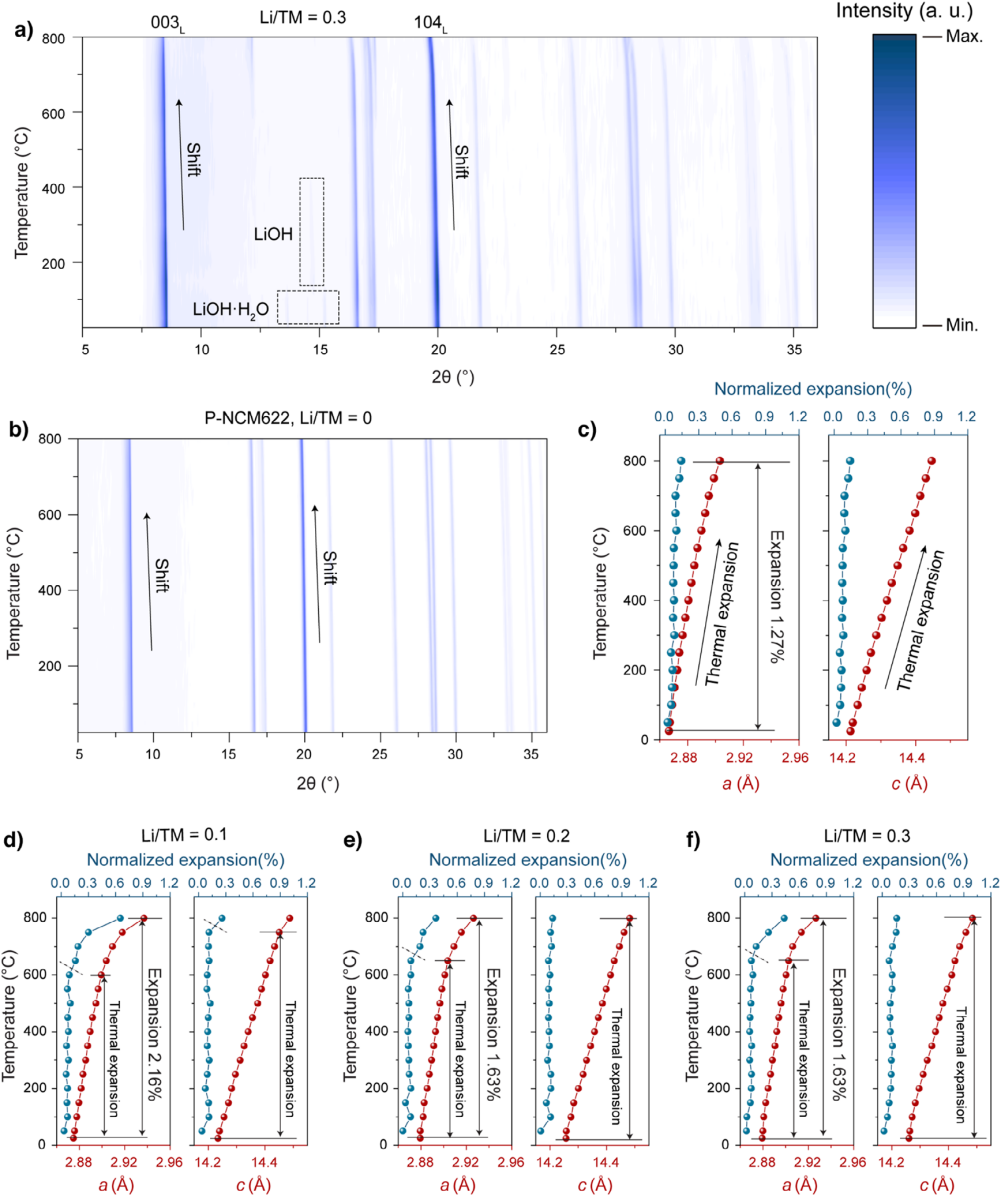
In situ high‐temperature XRD patterns (Mo‐Kα_1_ radiation, *λ* = 0.7093 Å) for a) Li/TM = 0.3 and b) P‐NCM622 without adding lithium salts. Corresponding calculated *a* and *c* and their normalized expansion for c) P‐NCM622, d) Li/TM = 0.1, e) Li/TM = 0.2, and f) Li/TM = 0.3 (the lattice expansion in the figure was normalized by calculating the growth of the lattice parameters over each 50 °C increment).

The structural evolution occurring during this reaction was initially studied by mixing S‐NCM622 with different Li/TM ratios (0.1, 0.2, and 0.3) and analyzing it using in situ high‐temperature XRD over a temperature range from room temperature (RT) to 800 °C under an air atmosphere. As shown in Figures 4a and S11, reflections corresponding to LiOH·H_2_O are detected below 150 °C, growing more prominent with increasing Li/M ratios. Around 150 °C, LiOH·H₂O loses water to form LiOH, which then melts around 450 °C (melting point of LiOH at ∼460 °C), indicated by the shift of reflections and their disappearance. Throughout the tested temperature range and various Li/M ratios, the layered structure of S‐NCM622 is maintained. However, a continuous shift toward lower angles is observed for 003_L_ and 104 _L_ reflections as the temperature increases (clearer view provided in Figure S12a–c), implying changes in lattice parameters. Such shifts of reflections have been noted in prior studies on direct recycling of layered materials,^[^ ^38^, ^39^, ^40^ ^]^ though the mechanisms may not have been fully explored. To comprehend this behavior, we further characterized both P‐NCM622 and S‐NCM622 without adding additional lithium source across the same temperature range (Figures 4b and S13). Cell parameters for all samples were then extracted using Rietveld refinement (Table S4–S8, the relatively high R_p_ and R_wp_ values observed in the samples are ascribed to the limited XRD data quality).

For P‐NCM622, the *a* and *c* values also expand as the temperature increases (Figure 4c). Notably, the expansion of *a* and *c* remain relatively constant over each 50 °C increment, suggesting that the expansions should be mainly ascribed to anharmonicities of lattice vibrations (thermal expansion). The differing thermal expansion of *a* and *c* can be explained by weaker interlayer forces (O‐Li‐O) than intralayer forces (O‐TM‐O). Yabuuchi et al.’s work on the thermal instability of cycled lithium‐nickel‐manganese oxides demonstrates similar conclusions.^[^ ^41^ ^]^ In case of S‐NCM622, similar expansion trends in *a* and *c* are found (Figure S14). Nevertheless, there is an evident increase in the expansion rate *a* at 650 °C, with gradual intensification as temperature further increases. The increases could indicate some degree of structural degradation, resulting from inferior thermal stability. Since *a* is generally linked to TM─O bonding distance, the higher expansion of *a* likely reflects a reduction in the oxidation states of TMs.^[^ ^42^, ^43^ ^]^ When lithium compensation is applied, the expansion behavior under Li/M = 0.1 closely resembles that of untreated spent NCM622 (Figure 4d). However, at higher lithium compensation ratios, the evident increase in the expansion rate of *a* is delayed to 700 °C, and the overall expansion is largely inhibited (Figure 4e,f). This phenomenon might result from the enhanced lithiation and structural repair promoted by higher lithium content, which results in improved thermal stability.

**Figure 5:**
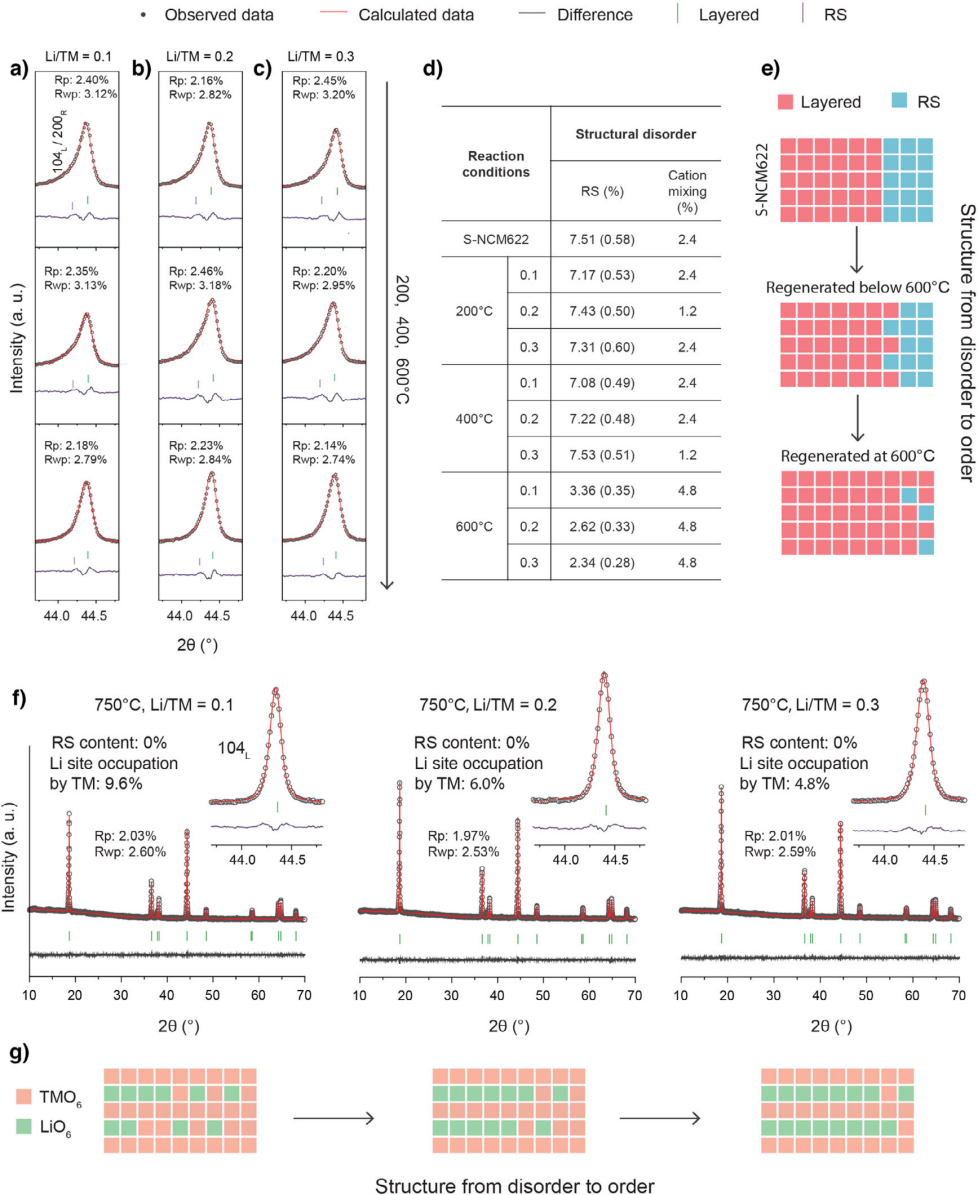
Ex situ XRD (Cu‐Kα_1_ radiation, *λ* = 1.54056 Å) and corresponding refinement results (focusing on 104_L_ reflection) for materials regenerated under different temperatures (200, 400, and 600 °C) with a Li/TM ratio of a) 0.1, b) 0.2, and c) 0.3. d) Corresponding calculated content of RS and cation mixing in these materials through Rietveld refinement. e) Schematic illustration for the structure from disorder to order when materials regenerated ≤ 600 °C. f) Ex situ XRD patterns and corresponding refinement results for materials regenerated at 750 °C with Li/TM ratio of 0.1, 0.2, and 0.3, respectively. (g) Schematic illustration for the structure from disorder to order when materials regenerated at 750 °C with different Li/TM ratios.

Due to the thermal expansion, it is challenging to fully understand the evolution of structural order during the repair process through in situ XRD findings. Figure 5a–f presents ex situ XRD results to further elucidate the structural evolution, where the samples were heated to different temperatures (200, 400, 600, 750, and 900 °C) with various Li/TM ratios (0.1, 0.2, and 0.3) and then cooled down to RT. Unlike the in situ findings, the ex situ results reveal that drastic lattice expansions are only observed at 900 °C (Figure S15), as indicated by the notable reflection shifts, which are related to structural degradation.

To better understand the changes below 750 °C, we conducted two‐phase Rietveld refinement (layered and RS), focusing on the structural disorder. Here, the structural disorder is believed to originate primarily from two sources: 1) the random distribution and mixing of cations within a local region, which alters the local crystal's symmetry and is referred to as RS‐type domain. In XRD, such disorder is mainly reflected in profile anomalies in reflections. 2) the occupation of Li octahedral sites by TM ions, which does not necessarily change the symmetry but still contributes to disorder, known as cation mixing. In XRD, this type of disorder is primarily reflected in the relative peak intensity.

For the materials regenerated below 600 °C, no significant reduction in structural disorder is observed compared to S‐NCM622, as indicated by similar levels of RS and cation mixing (Figure 5a–d). At 600 °C, however, the regenerated materials exhibit a noticeable alleviation in profile anomalies in reflections, corresponding to a significant decrease in RS content alongside a slight increase in cation mixing. These observations demonstrate an overall improvement in structural order. This transition from disorder to order, as illustrated in Figure 5e, can be attributed to the restoration of rhombohedral symmetry, driven by the reduction of domains with disordered RS‐type cation ordering. These findings account for the noticeable capacity recovery observed after 600 °C, which is ascribed to the obviously improved structural order. As the temperature reaches 750 °C, the profile anomalies in reflections nearly disappear, indicating the restoration of rhombohedral symmetry (Figure 5f). However, despite this recovery, the underlying disorder may still persist. This is evident in the varying degrees of TM occupation at Li octahedral sites among regenerated materials with different Li/TM ratios: 9.6%, 6.0%, and 4.8% for Li/TM ratios of 0.1, 0.2, and 0.3, respectively. Notably, the material regenerated with a Li/TM ratio of 0.1 exhibits significantly increased cation mixing, suggesting that structural order remains poor despite the decrease of RS‐type domains.

Therefore, it could be challenging to define whether the structure of materials regenerated at 750 °C with low Li/TM ratios is more ordered than those regenerated at 600 °C with high Li/TM ratios, based solely on the ex situ XRD results. However, it can be conclusively stated that, at 750 °C, the structural order of regenerated materials is strongly influenced by the Li/TM ratio. This also explains the significant impact of the Li/TM ratio on capacity recovery at 750 °C. The structural evolution from a Li/TM ratio of 0.1 to 0.3 at this temperature, as shown in Figure 5g, can be ascribed to the alleviation of the cation mixing, driven by the cation migration and rearrangement. This alleviation contributes to the improvement of capacity recovery. As such, R‐NCM622 (750 °C‐0.3) achieves the highest capacity among all regenerated materials, corresponding to the optimal structural order.

Overall, the combined in situ and ex situ XRD findings provide preliminary insights into the long‐range structural evolution during the repair process, highlighting a strong correlation between improved structural order and enhanced capacity recovery, driven by elevated temperature and Li/TM ratio.

**Figure 6:**
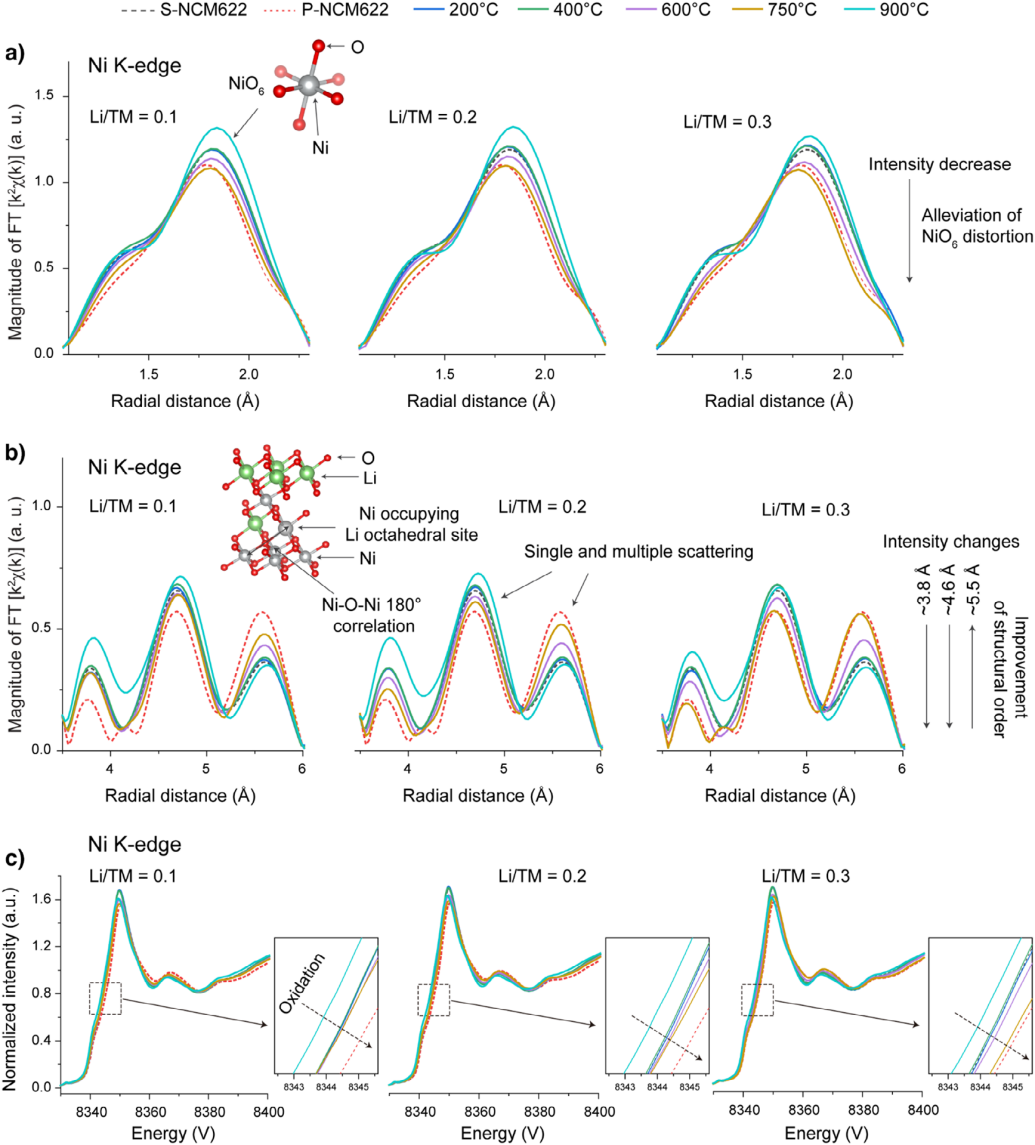
EXAFS results of Ni K‐edge a) from 1.1 to 2.3 Å, and b) from 3.5 to 6 Å, for materials regenerated under different temperatures (200, 400, 600, 750, and 900 °C) with various Li/TM ratios (0.1, 0.2, and 0.3). c) Normalized XANES spectra of Ni K‐edge for materials regenerated under different temperatures (200, 400, 600, 750, and 900 °C) with various Li/TM ratios (0.1, 0.2, and 0.3).

To probe deeper insights into the structural evolution during the repair process, particularly in differentiating the local structural changes among Ni, Co, and Mn, EXAFS analysis was conducted on the ex situ samples. In the Ni K‐edge spectra (Figure S16), the magnitude of the Ni‐O peak (∼1.8 Å) exhibits a clear temperature‐driven trend, largely independent of Li/TM ratios (Figure 6a). Specifically, the FT magnitude of Ni─O peak begins to decrease at 600 °C, reaches its minimum at 750 °C, and increases at 900 °C. This trend aligns well with the changes in profile anomalies in reflections observed in ex situ XRD. Although several factors—such as lattice distortions, static and thermal disorder, and interference from multiple scattering paths—can influence the FT magnitude, the observed decrease is believed to closely correlate with the recovery of rhombohedral symmetry, as supported by the consistency between ex situ XRD and EXAFS results. More fundamentally, this decrease may stem from changes in the CCP oxygen framework of the NCM622 material, which results in reduced O backscattering to Ni. On the one hand, the reverse transition from Ni^2+^‐O_6_ to Jahn–Teller active Ni^3+^‐O_6_ could restore the oxygen framework closer to its original state. Alternatively, the more dispersed distribution of Ni^2+^ within the Li layer—due to cation rearrangement, lithium intercalation, and TM migration from the Li layer back to the TM layer—might mitigate oxygen framework distortions induced by structural degradation and similarly contribute to restoring the oxygen framework to its original state. In contrast, the Co and Mn K‐edge spectra reveal much smaller changes in their Co/Mn‐O peaks across different temperatures and Li/TM ratios (Figures S17 and S18). These observations indicate that the structural repair associated with the recovery of rhombohedral symmetry should be mainly ascribed to the changes in NiO_6_ octahedral structure. In other words, the migration of Ni from the Li layer back to the TM layer dominate the structural repair.

Moreover, changes in FT magnitude at ∼3.8, ∼4.6, and ∼5.5 Å in the Ni spectra, which reflect the longer‐range structure, to some extent provide a clearer view into the relative amounts of TM ions (mainly Ni) within the Li layer. As observed, evident FT magnitude changes begin at 600 °C, indicating a clear decrease in TM ions within the Li layer. This can correspond to an obvious improvement in structural order, consistent with ex situ XRD observations. It should be mentioned that, as the number of TM ions within the Li layer changes, the FT magnitude variations at ∼3.8 and ∼4.6 Å, in some cases, are less pronounced than those at ∼5.5 Å. This may be attributed to the greater complexity of interlayer coordination environments compared to those of intralayer. Additionally, the Li/TM ratio also plays a crucial role in influencing FT magnitude when temperatures are above 600 °C, particularly at 750 °C. This suggests that a higher Li/TM ratio can also enhance structural order by promoting the migration of TM ions back to the TM layer, but mainly after 600 °C. It is worth noting that, although the ex situ XRD results show incomplete recovery of rhombohedral symmetry in materials regenerated at 600 °C with Li/TM ratios of 0.2 and 0.3, compared to the material regenerated at 750 °C with a Li/TM ratio of 0.1, the EXAFS results likely indicate a similar amount of TM ions within the Li layer across these samples (Figure S19). Consequently, it is observed that their initial capacities are quite comparable (Figure S20). These observations highlight that the improved capacity is more likely associated with the decrease in the amount of TM ions within the Li layer. Lastly, all TM spectra reveal that the peak shapes and intensities for R‐NCM622 closely resemble those of P‐NCM622 (Figure S21). This similarity confirms the successful restoration of the layered structure, consistent with the ex situ XRD results.

Additionally, such TM migration from the Li layer back to the TM layer would highly rely on their oxidation. This is because the TM migration requires their oxidation to supply electrons to O_2_, forming O^2−^ and realizing the expansion of CCP oxygen framework. This structural adjustment results in the formation of new octahedral site, which is essential for Li insertion and TM migration. As such, we followed the structural analysis with the chemical‐state analysis of TMs in the ex situ samples using XANES results. In the Ni K‐edge spectra (Figure 6c), it is observed a notable edge shift toward higher energy beginning around 600 °C and peaking at 750 °C, indicative of Ni oxidation. Below 600 °C, the Ni oxidation remains relatively unaffected by the Li/TM ratios. Yet, as the temperature increases, differences in Li/TM ratios significantly impact the Ni oxidation, especially at 750 °C. At 900 °C, the edge shifts back toward lower energy, suggesting a reduction in the Ni oxidation state. These redox changes align closely with the magnitude variations observed at ∼3.8, ∼4.6, and ∼5.5 Å in the Ni spectra, reinforcing the strong correlation between TM migration and their oxidation.

For Co and Mn K‐edge XANES spectra, more subtle edge shifts are observed but follow a similar overall trend as Ni (Figures S22 and S23). Importantly, it is observed that the Co and Mn edges shift toward higher energy at lower temperatures than Ni and they experience relatively smaller edge shifts at 900 °C. These trends likely suggest that Co and Mn undergo slight oxidation already at lower temperatures and experience less reduction at elevated temperatures, compared to Ni. Although the EXAFS results do not directly show Co and Mn migration, such edge shifts at lower temperature appear to indicate a preferential onset of migration of Co and Mn from the Li layer to the TM layer.^[^ ^44^ ^]^ Overall, these observations suggest that the TM migration from the Li layer to the TM layer, accompanied by their oxidation and re‐lithiation, would serve as the primary mechanism for structural repair.

**Figure 7:**
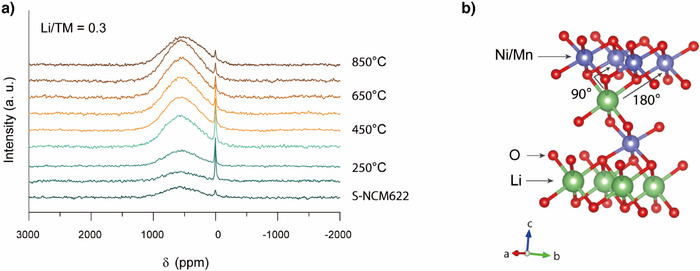
a) ^6^Li solid‐state NMR spectra for materials regenerated under different temperatures (150–850 °C, per 100 °C) with a Li/TM ratio of 0.3. b) The local environment in the TM/Li layers that gives rise to the isotropic resonance at ∼550 ppm is shown. The interactions occur via the oxygen as intermediate atom.

To fully elucidate the mechanisms underlying the structural repair process, it is equally crucial to understand the coordination environment of re‐intercalated lithium. Figure 7a presents the ex situ ^6^Li solid‐state nuclear magnetic resonance (NMR) spectra of S‐NCM622 mixed with ^6^LiOH·H_2_O at a Li/TM ratio of 0.3, sintered at different temperatures (150–850 °C, per 100 °C), and then annealed to RT. The “S‐NCM622” sample refers to spent material without added ^6^LiOH·H_2_O. Two major cluster groups of resonances are observed: one at ∼550 ppm and one at ∼0 ppm. The higher frequency resonances (∼550 ppm) are assigned to Li local environments in Li layers, resulting from the interactions between the Li nucleus and unpaired electron spin density of Ni and Mn ions in the first and second cation coordination shell, as shown in Figure 7b.^[^ ^45^, ^46^ ^]^ The lower‐frequency resonances (∼0 ppm) are attributed to residual lithium salts on the surface.

In the spectra, although S‐NCM622 does not contain added ^6^Li, there are still small contributions to the signal, corresponding to the natural isotope composition of Li (93% ^7^Li, 7% ^6^Li ^[^ ^47^ ^]^). The intensity of the high‐frequency resonances begins to notably increase as the temperature reaches 250 °C, accompanied by a sharp decrease in the low‐frequency resonances, indicating the onset of lithiation. Besides, this phenomenon suggests that the lithiation initiates before the lithium salt transitions to a molten state. Between 250 and 750 °C, the high‐frequency resonances progressively intensify and sharpen, while the low‐frequency resonances decrease, reflecting ongoing lithiation (Figure S24, offering a clearer view). ICP‐OES and CGHE results show that the stoichiometry of R‐NCM622 is Li_1.003_Ni_0.600_Co_0.200_Mn_0.200_O_2.039_ (Table S9), indicating a recovery of Li and O. Yet, at 850 °C, the high‐frequency resonances exhibit a reversal, indicating lithium loss. According to ICP‐OES and CGHE results, the stoichiometry of the material regenerated at 800 °C with a Li/TM ratio of 0.3 is found to be Li_0.975_Ni_0.596_Co_0.201_Mn_0.203_O_1.982_ (Table S10), suggesting lithium and oxygen loss after 750 °C, consistent with the NMR findings. In addition, no other significant resonances are detected during this process, suggesting that the intercalated Li predominantly occupies octahedral sites and exists within a Li local environment.

Based on the XRD, XAS, and NMR findings, a clear relationship emerges among capacity recovery, phase transition, TM migration and oxidation, and re‐lithiation. In essence, a lower concentration of TM ions (mainly Ni) in the Li layer corresponds to a higher TM oxidation state and a more ordered structure, and thus improved capacity recovery. Overall, this structural repair process can be mainly divided into three stages:

**Stage I (below 600 °C)**: In this stage, the thermal energy is insufficient to significantly drive the oxidation state and interlayer migration of TMs, particularly for Ni, resulting in slow reaction kinetics. This is evident from the ex situ XRD data, which shows that the rhombohedral symmetry is not significantly recovered below 600 °C, and from the ex situ EXAFS and XANES results, which indicate no obvious TM migration or oxidation. Accordingly, the capacity recovery of the regenerated materials during this stage remains limited.

**Stage II (600–750 °C)**: This is the main stage for the transformation from RS to layered phase, where both temperature and lithium ratio play crucial roles in facilitating the cation migration and structural order. This is evidenced by the clear recovery of rhombohedral symmetry, along with noticeable TM migration and oxidation observed through XRD, EXAFS, and XANES analyses. As a result, the majority of the capacity recovery occurs during this stage.

**Stage III (above 750 °C)**: At high temperatures, Li/O release from the material could be triggered, undermining the repair effects and even inducing further structural degradation. This is also reflected in the XRD, XAS, and NMR results. Consequently, there is a continuous decline in capacity beyond 750 °C.

### Possible Thermal Solid‐state Reaction Mechanism

**Figure 8:**
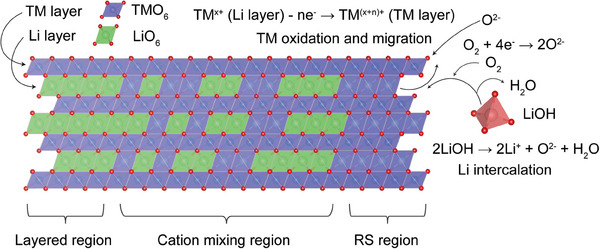
Schematic illustration for a possible thermal solid‐state reaction mechanism during the structural repair.

It is well established that the core mechanism of a thermal solid‐state reaction is driven by kinetic processes involving the cleavage and formation of chemical bonds.^[^ ^48^ ^]^ Heat plays a crucial role by supplying the energy required to overcome activation barriers, thereby facilitating ionic diffusion within the solid state.^[^ ^49^ ^]^ Based on the present findings, the solid‐state reaction driving the structural repair process can be summarized as follows: With the gradual application of heat, the material's energy increases, leading to the oxidation of TM ions within the Li layer. During this process, electrons from the TM ions are captured by O_2_, forming O^2^⁻ and generating new octahedral site in the TM layer. Subsequently, the oxidized TM ions migrate from the Li layer to the TM layer, while Li ions supplied by LiOH occupy the vacant octahedral sites in the Li layer, completing the insertion process. As TM ions continue to migrate and Li ions are inserted, cation diffusion along the *a*/*b* axis takes place, accompanied by cation rearrangement. This leads to the formation of a lithium‐ion concentration gradient in the Li layer along *a*/*b* axis within a localized region. This process is schematically illustrated in Figure 8.

During this reaction, heat emerges as the most critical factor in overcoming the activation energy required for TM migration and oxidation. EXAFS and XANES results suggest that Ni has the highest migration barrier from the Li layer to the TM layer compared to Co and Mn. Although Co and Mn may exhibit an earlier onset of migration, the hindered Li insertion and diffusion—due to the significant presence of Ni within the Li layer—may still slow their migration. As a result, noticeable migration of Ni, Co, and Mn is observed mainly after 600 °C, once the Ni migration barrier is overcome. After that, the Li/TM ratio plays an increasingly pivotal role in facilitating TM migration and ionic diffusion within the solid state, further enhancing structural order. This influence is likely tied to the concentration of Li at the solid‐contact surfaces and the resulting lithium‐ion concentration gradient within the metal oxides. In other words, a higher Li/TM ratio might contribute to reducing the TM migration barrier from the Li layer to the TM layer. Although higher Li/TM ratios are beneficial for restoring the structural order, their effect is not without limitations. As observed, higher Li/TM ratios (e.g., 0.4, 0.5, and 0.6) at 750 °C do not lead to further improvements in electrochemical capacity. However, these elevated ratios aid in achieving optimal structural order at slightly lower temperatures, as supported by the data presented in Figure S25.

### Potential Challenges for Direct Recycling

In this study, the structural repair of the spent NCM622 (retired after thousands of cycles with a capacity retention of 81%) is successfully accomplished, achieving structural order and initial capacity comparable to commercial NCM622.

By combining XRD, XAS, and NMR analyses, we extend our investigation beyond phase transitions to examine structural changes at an atomic level, providing valuable insights into cation migration and diffusion during the repair process. Notably, Ni migration from the Li layer back to the TM layer could be the greatest challenge in structural repair. Therefore, developing strategies to lower the Ni migration barrier might contribute to enhancing the electrochemical properties of regenerated material or reducing energy consumption during the repair process.

Moreover, it is observed that the regenerated material falls short of achieving satisfactory cycling performance when compared to commercial NCM622. This limitation may be attributed to the lack of additional modification strategies, such as element doping or surface coating. Alternatively, this limitation is likely due to potential impurities introduced during the pre‐treatment process (e.g., Al, F, and P) and the presence of unrecovered particle cracks in the regenerated material (Figure S26). Therefore, future research aimed at identifying the root causes of the poor cycling performance and developing strategies to address these challenges could be crucial for advancing sustainable battery recycling technologies.

## Conclusion

In summary, this study systematically investigates the thermal solid‐state structural repair process of spent NCM622 cathode material. Through multiscale characterization techniques, including XRD, XAS, and ^6^Li solid‐state NMR, the structural degradation in spent NCM622 and the long‐ and short‐range structural changes during the repair process are thoroughly examined. The findings identify Ni migration from the TM layer to the Li layer along with lithium and oxygen loss, reductions in Ni oxidation states, and local destruction in rhombohedral symmetry as the primary factors driving the structural degradation of NCM622.

During the repair process, both temperature and lithium compensation ratio are shown to play crucial roles in migration of TMs, particularly Ni, back to the TM layer and enhancing ionic diffusion within the solid state, thereby restoring the structural order in the spent material. Moreover, capacity recovery studies reveal a strong correlation between the restoration of structural order and the recovery of electrochemical performance. The regenerated material with optimal structural order (750 °C, Li/TM = 0.3) demonstrates a structural order and an initial capacity comparable to those of pristine NCM622, along with a 76% capacity retention after 150 cycles at 1 C charge/discharge over 3.0–4.3 V, validating the effectiveness of the repair process.

These findings highlight the potential of thermal solid‐state repair as a viable strategy for restoring the functionality of spent cathode materials, offering insights for future advancements in battery recycling and sustainability.

## Conflict of Interests

The authors declare no conflict of interest.

## Supporting information



Supporting Information

## Data Availability

The data that support the findings of this study are available from the corresponding author upon reasonable request.
