# Supplementary material for: Insights into the Mechanisms Behind Structural Repair of Spent Layered Cathode Materials for Lithium‐Ion Batteries
Source: Angew Chem Int Ed Engl. 2025 Jun 22;64(32):e202504382. doi: 10.1002/anie.202504382 (PMC12322646; doi:10.1002/anie.202504382)
Supplement: Supplementary file 1 — Supporting Information [file ANIE-64-e202504382-s001.docx]

**Supporting Information**

**Insights into the Mechanisms Behind Structural Repair of Spent Layered Cathode Materials for Lithium-Ion Batteries**

Shuaiwei Liu ^a^, Hao Liu ^a^, Arseniy Bokov ^a, b^, Mohammad Jaleh ^a^, Hang Li ^a^, Sylvio Indris ^a, c^, Oleksandr Dolotko ^a, b^, Aleksandr Kalinko ^d^, Edgar Eduardo Villalobos Portillo ^e^, Carlo Marini ^e^, Thomas Bergfeldt ^a^, Michael Knapp ^a, *^, Helmut Ehrenberg ^a, b^

*^a^ Karlsruhe Institute of Technology (KIT), Institute for Applied Materials (IAM), Hermann-von-Helmholtz-Platz 1, D-76344 Eggenstein-Leopoldshafen, Karlsruhe, Germany.*

*^b^ Helmholtz-Institute Ulm for Electrochemical Energy Storage (HIU), P.O. Box 3640, D-76021 Karlsruhe, Germany.*

*^c^ Applied Chemistry and Engineering Research Centre of Excellence (ACER CoE), Université Mohammed VI Polytechnique (UM6P), Lot 660, Hay Moulay Rachid, Ben Guerir, 43150, Morocco*

*^d^ Deutsches Elektronen-Synchrotron (DESY), Notkestrasse 85, Hamburg, Germany.*

*^e^ CELLS-ALBA, Synchrotron, Barcelona E-08290, Spain.*

* **Corresponding author at:** Karlsruhe Institute of Technology (KIT), Institute for Applied Materials (IAM), Hermann-von-Helmholtz-Platz 1, D-76344 Eggenstein-Leopoldshafen, Karlsruhe, Germany.

**E-mail address:** [michael.knapp@kit.edu](mailto:michael.knapp@kit.edu)

**Methods**

**Production and Charge/discharge cycling of pouch cell for generation of spent material.** The pouch cells were provided by the KIT Battery Technology Center (KIT-BATEC) ^1^. The cathode with aluminum foil as current collector was composed of NCM622, polyvinylidene fluoride (PVDF) binder, and conductive carbon additive in a weight ratio of 96.0 : 2.0 : 2.0. The anode with copper foil as current collector contained graphite, binder consisting of sodium carboxymethylcellulose and styrene-butadiene rubber latex (CMC/SBR), and conductive additive carbon black in a weight ratio of 96.0 : 2.8 : 1.2. The cathode and anode along with ceramic coated polyethylene terephthalate fabric (CC-PET) as a separator, were assembled using 1M LiPF_6_ in EC, DMC and VC as additive in the electrolyte to build multi-layer pouch cells, resulting in a nominal capacity of 12 Ah. The cells underwent 2100 cycles at a 1 C charge and discharge rate within the voltage range of 3.0-4.2 V until their capacity reached approximately 81 % of their initial level, indicating the end of their useful life. Subsequently, the LIBs were deeply discharged to 0 V, short-circuited, and disassembled to extract the cycled cathode and anode.

**Direct regeneration of cathode active material**. The as-obtained cathode sheets were cut into small pieces, and heated at 450°C for 4 hours under an air atmosphere to decompose the PVDF binder, thereby facilitating the delamination of cathode active material (CAM) from aluminum foil. After that, CAM was collected by manually shaking the cathode sheets, yielding approximately 65 g spent NCM622 powder ^2^. Note that ICP-OES analysis indicates an Al impurity (0.3-0.5 *wt*%) in this powder. In addition, X-ray photoelectron spectroscopy (XPS) measurement detects the presence of F (~8.3 *at*%) and P (~2.2 *at*%) impurities besides the Al impurity (~4.6 *at*%), along with significant oxygen-containing lithium salts (e.g., LiOH and Li_2_CO_3_) in this powder.

The spent NCM622 was regenerated by a thermal solid-state reaction with a subsequent washing step, and a second sintering process to remove the residual lithium salts on the surface. Specifically, the powder was mixed homogeneously by mortar with LiOH·H_2_O (Sigma Aldrich, >98 %) in different lithium compensation/TM molar ratios of 0.1/1.0, 0.2/1.0, and 0.3/1.0. The obtained mixture was heated under an air atmosphere in a muffle furnace (100-900°C for 12 h, heating rate 5°C min^-1^ and passive cool down to room temperature). During the second sintering process after washing, materials regenerated below 650°C underwent sintering at the same temperature as the initial sintering, with a holding time of 6 hours. In contrast, materials regenerated above 650°C were subjected to a second sintering at 650°C, also with a 6-hour holding time.

**Electrode Preparation**. The electrodes were fabricated by thoroughly blending the active material, Super C65 conductive carbon black (MTI Co., Ltd.), and polyvinylidene fluoride (PVDF, Sigma-Aldrich) in an 8:1:1 mass ratio using a mortar. N-methyl-2-pyrrolidone (NMP) with a moisture content below 0.1% (VMR) served as the solvent. The mixture was homogenized using a planetary mixer (THINKY ARV-310) at 2000 rpm for 10 minutes in an air atmosphere. The resulting slurry was uniformly applied onto 15 *μ*m thick aluminum foil (Häberle LABORTECHNIK GmbH & Co.KG) using a ZUA 2000 Universal applicator at a speed of 25.0 mm/s, achieving a coating thickness of 150 μm. The coated aluminum foil was dried at 80°C in ambient conditions for 6 hours to remove the NMP solvent. After drying, the electrode material was punched into 12 mm diameter disks (with an approximate active material loading of 3 mg/cm²) using a handheld punch (NOGAMIGIKEN Co., Ltd.). A final drying step was performed in a Büchi glass oven (B-585) under vacuum at 120°C for 12 hours to ensure complete solvent removal.

**Cell Assembly and Electrochemical Characterization**. To assess the electrochemical performance, 2032-type coin cells were assembled. Each cell consisted of a lithium metal anode (14 mm diameter, 0.25 mm thickness, PI-KEM), a WHATMAN GF/C membrane (WHATMAN INT. LTD, Chro.) serving as the separator, and 100 μL of electrolyte. The electrolyte (Sigma-Aldrich) comprised 1 M lithium hexafluorophosphate (LiPF₆) dissolved in a 1:1:1 volume ratio of ethylene carbonate (EC), diethyl carbonate (DEC), and dimethyl carbonate (DMC), with the moisture content maintained below 10 ppm. All assembly processes were conducted in an argon-filled glovebox with O₂ and H₂O levels kept below 0.1 ppm. Electrochemical cycling and rate test were performed at a constant temperature of 25°C using a Biologic VMP3 multichannel battery testing system, operating within a voltage window of 3.0–4.3 V. To ensure reproducibility, all electrochemical experiments were conducted using a minimum of two-coin cells per test. The current density for each cell was determined based on the mass of the active material in the electrode, ensuring accurate and consistent comparisons across measurements.

**Materials characterization**. The elements except oxygen of the materials were analyzed by Inductively Coupled Plasma Optical Emission Spectrometry (ICP-OES), and oxygen was analyzed by Carrier gas hot extraction (CGHE).

The ex-situ X-ray Diffraction (XRD) experiments were conducted on a STOE Stadi P powder diffractometer with monochromatic Cu-Kα_1_ radiation (λ = 1.54056 Å) in transmission geometry. The measurements were performed at room temperature with a 0.015° 2θ step between 10 and 70 degrees of 2θ. The Kapton film's presence visibly adds an amorphous-like background in the XRD patterns at 10° < 2θ < 17°.

The in-situ high-temperature XRD experiments were conducted on a STOE Stadi P powder diffractometer with monochromatic Mo-Kα_1_ radiation (λ = 0.7093 Å). The samples were loaded into quartz capillaries with an outer diameter of 0.5 mm. The tests started at room temperature, followed by a 1-hour dwell at every 50°C increment. XRD data collection was performed during the dwell period, taking approximately 40 minutes.

The Synchrotron Radiation Diffraction (SRD) data were sequentially collected alongside Hard X-ray absorption Spectroscopy (XAS) at BL16 (NOTOS) at ALBA (Spain) with a synchrotron radiation energy of 13 keV. Specifically, the XAS data were acquired first, with each edge requiring approximately 5 minutes. Subsequently, the system switched to SRD mode, where SRD data collection was completed in approximately 30 seconds.

The Rietveld refinement based on the obtained XRD and SRD patterns was performed in the FullProf software package ^3^. The background of all the diffraction patterns was fitted using linear interpolation between selected data points in regions with no reflections present. Thompson-Cox-Hastings pseudo-Voigt function was used for the reflection profile shape description. The scale factor, lattice parameter, fractional coordinates of atoms, their overall isotropic displacement (temperature) parameter, zero angular shift, profile shape parameters, and half-width (Caglioti) parameters were allowed to vary during fitting.

The ex-situ Hard X-ray Absorption Spectroscopy (XAS) experiments, focusing on collecting Extended X-ray Absorption Fine Structure (EXAFS) spectra, were performed at beamline P64 Advanced X-ray Absorption Spectroscopy at PETRA III (Germany). The obtained XAS data were processed using ATHENA software ^4^. The corresponding k^2^χ(k) data are presented in **Fig. S27-29**, with k-ranges for Fourier transformation selected as 3-11, 3-10.3 and 3-10.5 Å^-1^ for Ni, Co and Mn K-edge, respectively. During Fourier transformation processing in ATHENA, phase correction was applied, resulting in an ~3 Å shift.

Solid-state nuclear magnetic resonance (NMR) spectroscopy was conducted on Bruker Avance 200 MHz spectrometer at a magnetic field of 4.7 T. The spectra were acquired with 1.3 mm rotors at a spinning speed of 60 kHz. The recycle delay was set to 1 s, and the Larmor frequency was 29.5 MHz for ^6^Li. ^6^Li NMR spectra were measured using a rotor-synchronized Hahn-echo pulse sequence (90°−τ–180°−τ–acquisition) with a 90° pulse length of 0.95 *μ*s. The ^6^Li NMR shifts were referenced using an aqueous 1 M ^6^LiCl solution (0 ppm). All spectra were normalized with respect to sample mass and number of scans. The temperatures of the rotors during spinning were investigated using the shift of ^207^Pb in Pb(NO_3_)_2_ ^5, 6^.

**Figures**

**Fig. S1.** Electrochemical performances for pristine NCM

**Fig. S2.** One-phase Rietveld refinement for pristine and spent NCM

**Fig. S3.** Bragg reflections of standard NiO

**Fig. S4**. SRD patterns and refinement results for pristine and spent NCM

**Fig. S5.** Schematic illustration for RS-type and NCM layered structure

**Fig. S6.** Schematic illustration for structural degradation in NCM materials

**Fig. S7.** XANES of Ni K-edge for spent and pristine NCM

**Fig. S8.** Schematic illustration of Ni-O-Ni 180° correlation

**Fig. S9.** Initial charge/discharge capacities for S-NCM622 with and without water washing

**Fig. S10.** Cycling performance of NCM regenerated at 750°C

**Fig. S11.** High-temperature XRD Li/TM = 0.1 and Li/TM = 0.2

**Fig. S12.** Enlarged high-temperature XRD patterns for Li/TM of 0.1-0.3

**Fig. S13.** High-temperature XRD for spent NCM

**Fig. S14.** Calculated lattice parameters from high-temperature XRD for spent NCM

**Fig. S15.** Ex-situ XRD for NMC regenerated at different conditions

**Fig. S16**. EXAFS spectra of Ni K-edge for NCM regenerated at different conditions

**Fig. S17**. EXAFS spectra of Co K-edge for NCM regenerated at different conditions

**Fig. S18**. EXAFS spectra of Mn K-edge for NCM regenerated at different conditions

**Fig. S19**. Enlarged region of EXAFS spectra of Ni K-edge for NCM regenerated at different conditions

**Fig. S20.** Discharge capacity for NCM regenerated at different conditions

**Fig. S21.** EXAFS spectra of Ni/Co/Mn K-edge for pristine/spent/regenerated NCM

**Fig. S22**. XANES of Co K-edge NCM regenerated at different conditions

**Fig. S23.** XANES of Mn K-edge NCM regenerated at different conditions

**Fig. S24.** Solid state ^6^Li NMR for NCM regenerated at different conditions

**Fig. S25.** Cycling performances for NCM regenerated at 700°C and 750°C

**Fig. S26.** SEM images of R-NCM622 (a-b) before and (c-d) after 200 cycles

**Fig. S27.** k^2^χ(k) data of Ni K-edge

**Fig. S28.** k^2^χ(k) data of Co K-edge

**Fig. S29.** k^2^χ(k) data of Mn K-edge

**Tables**

**Table S1.** ICP-OES and CGHE analyses for spent NCM without washing

**Table S2.** ICP-OES and CGHE analyses for spent NCM with washing

**Table S3.** ICP-OES and CGHE analyses for pristine NCM

**Table S4.** Cell parameters from in-situ high-temperature XRD of Li/TM=0.1

**Table S5.** Cell parameters from in-situ high-temperature XRD of Li/TM=0.2

**Table S6.** Cell parameters from in-situ high-temperature XRD of Li/TM=0.3

**Table S7.** Cell parameters from in-situ high-temperature XRD of pristine NCM

**Table S8.** Cell parameters from in-situ high-temperature XRD of spent NCM

**Table S9.** ICP-OES and CGHE analyses for material regenerated at 750℃ with Li/TM of 0.3

**Table S10.** ICP-OES and CGHE analyses for material regenerated at 800℃ with Li/TM of 0.3


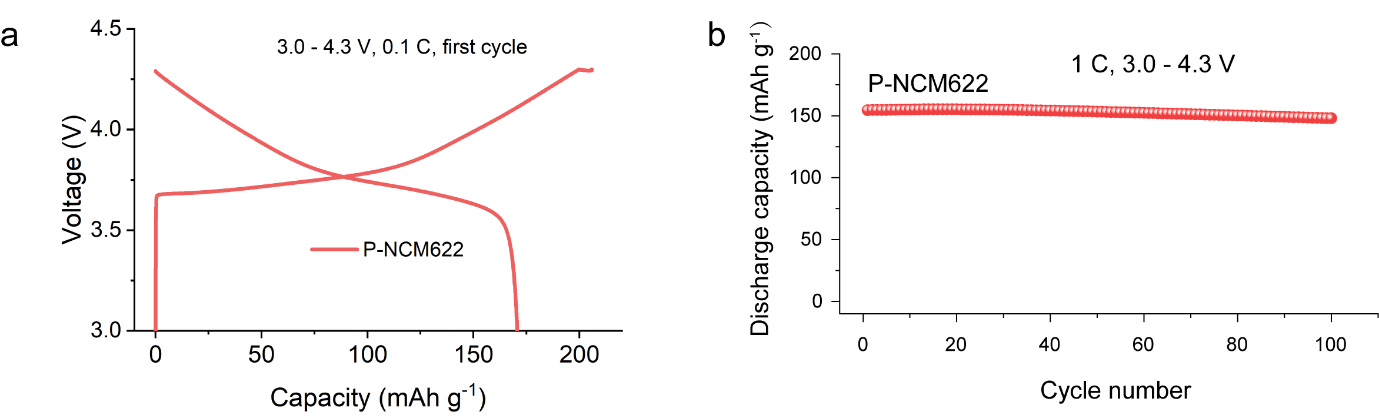


**Fig. S1.** (a) Initial charge/discharge capacities at 0.1C over 3.0-4.3 V and (b) cycling performance at charge-discharge rate of 1 C within the same voltage range for P-NCM622.


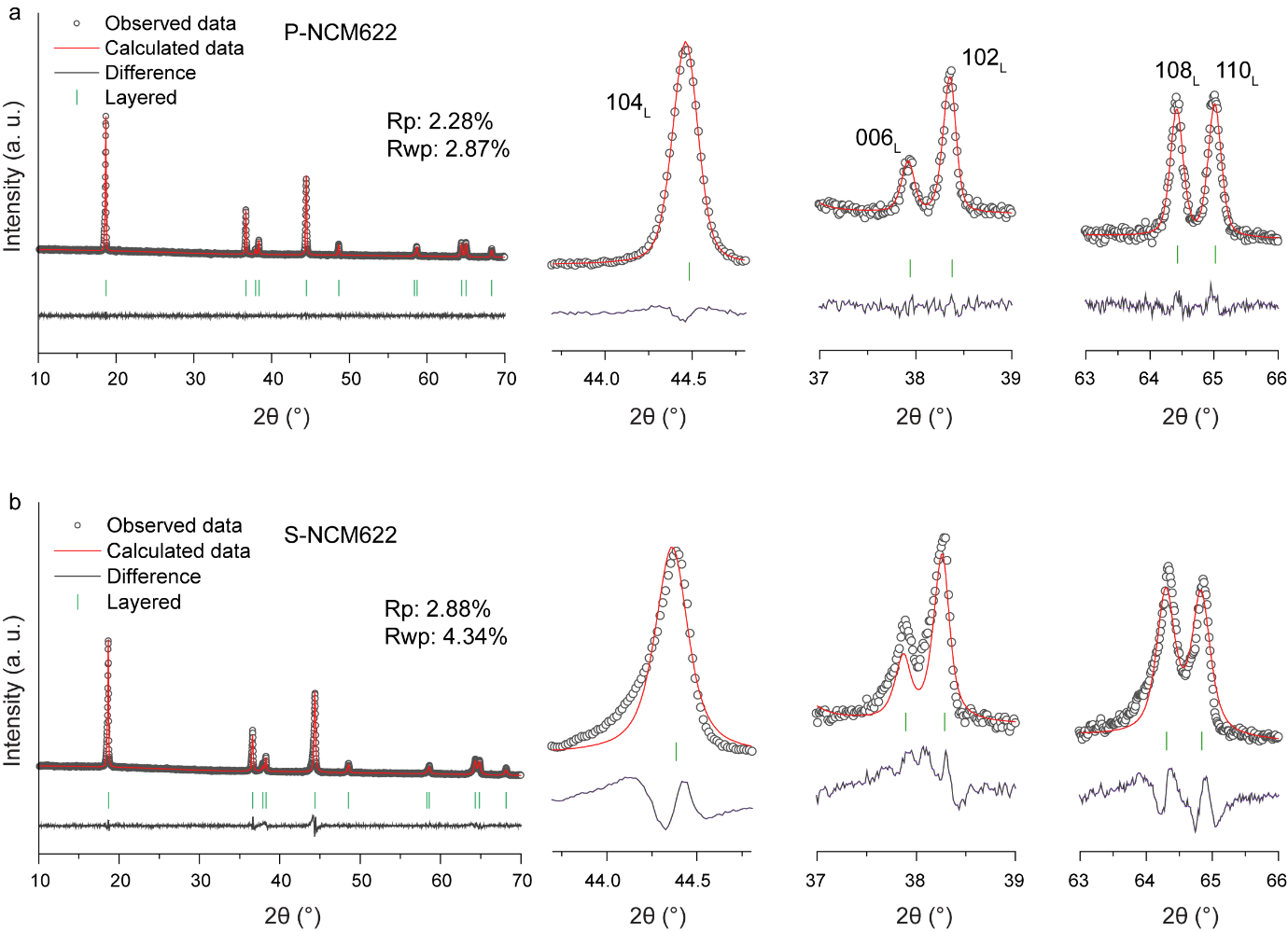


**Fig. S2.** XRD patterns and corresponding one-phase (layered) refinement results for (a) pristine NCM622 (P-NCM622) and (b) spent NCM622 (S-NCM622).


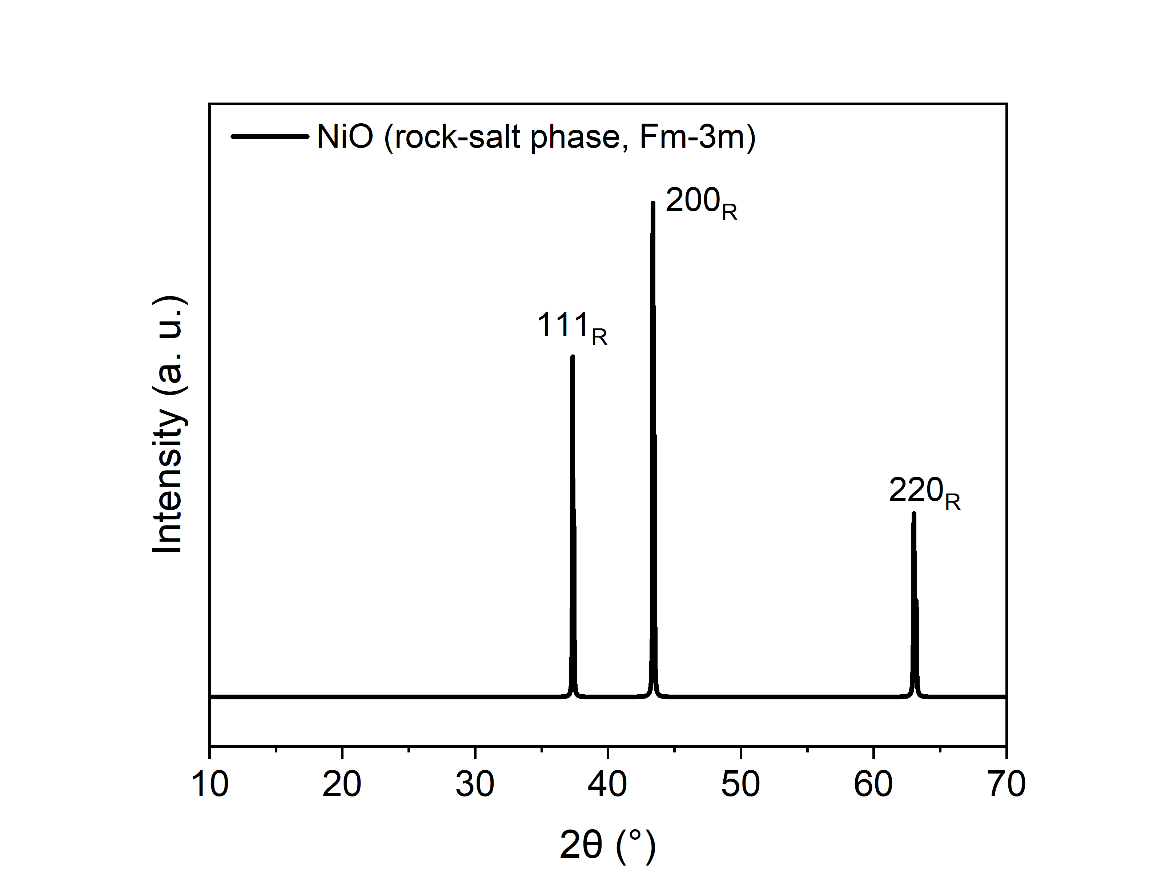


**Fig. S3.** Bragg reflections of standard NiO (rock-salt phase, *Fm-3m*).


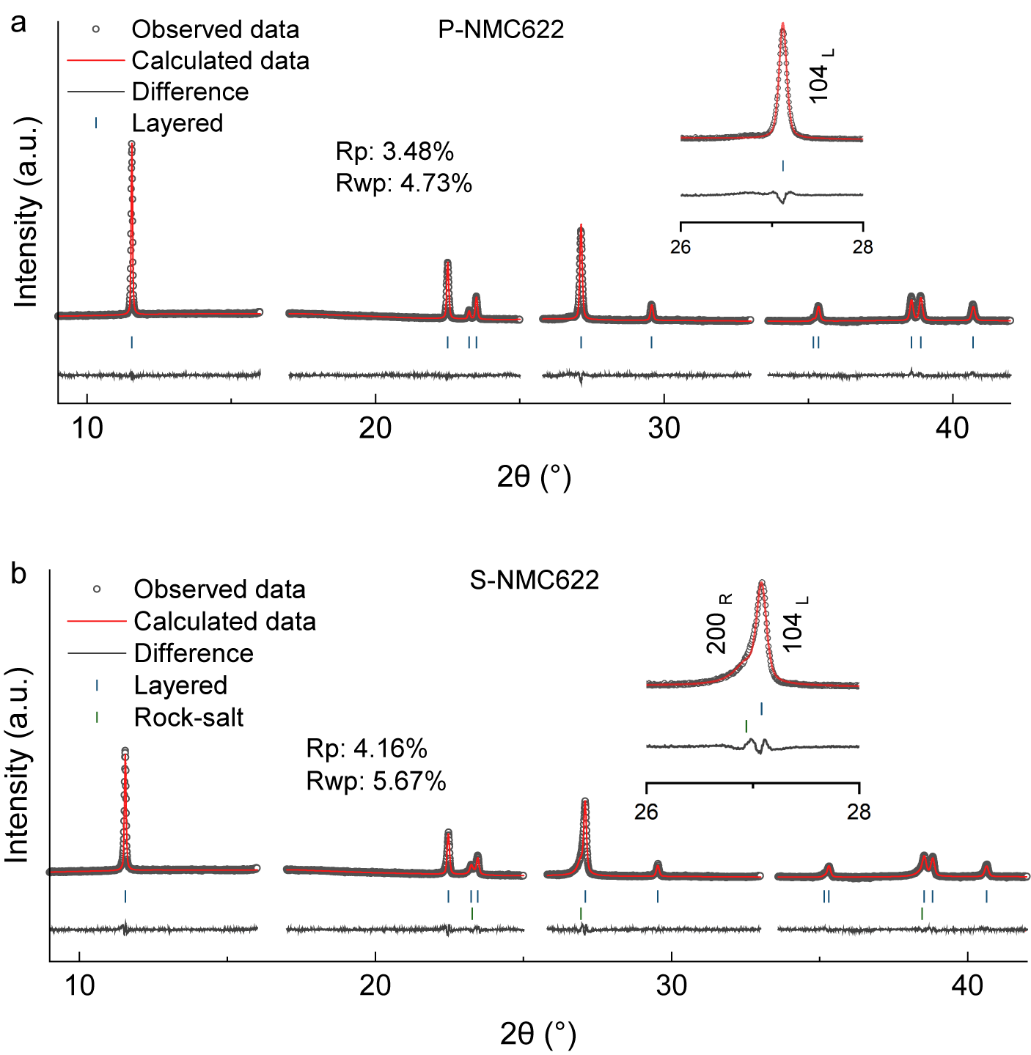


**Fig. S4.** Synchrotron Radiation Diffraction (SRD) patterns (λ = 0.9542 Å) and corresponding refinement results for (a) pristine NCM622 (P-NCM622) and (b) spent NCM622 (S-NCM622).


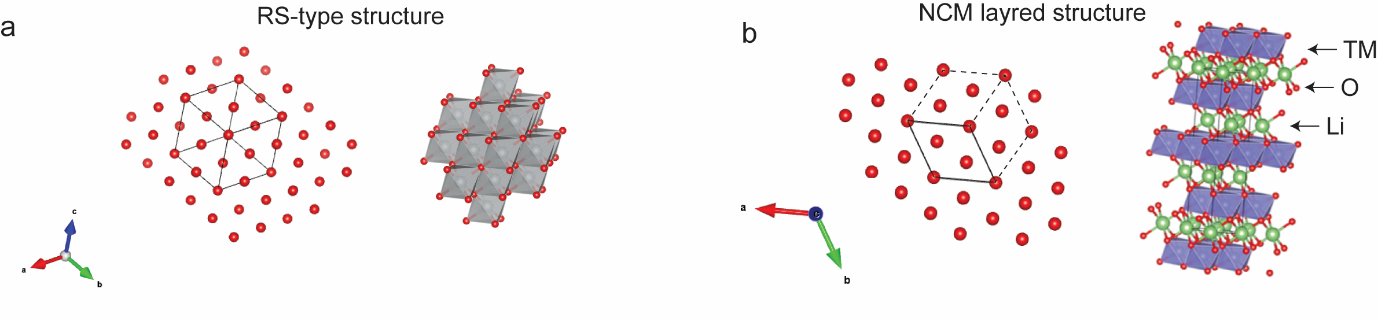


**Fig. S5.** Schematic illustration for (a) RS-type structure and (b) NCM layered structure.


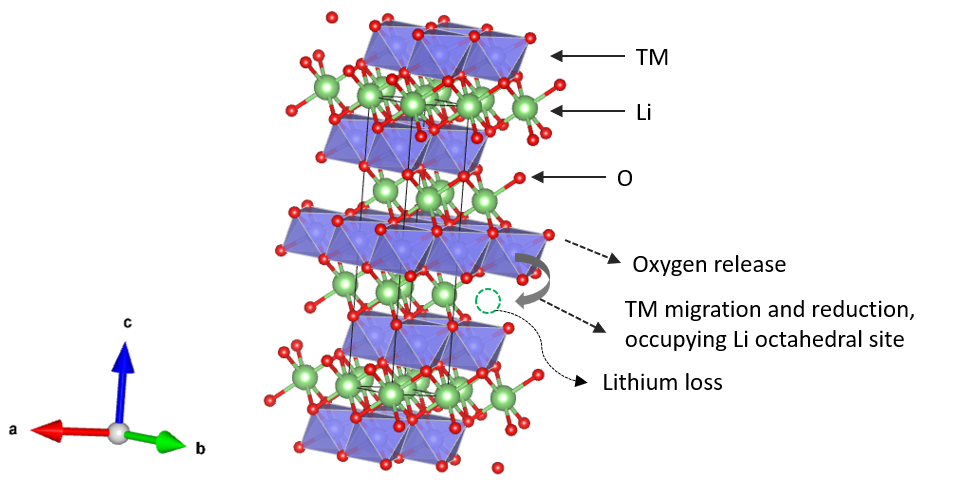


**Fig. S6.** Schematic illustration for structural degradation in NCM materials.


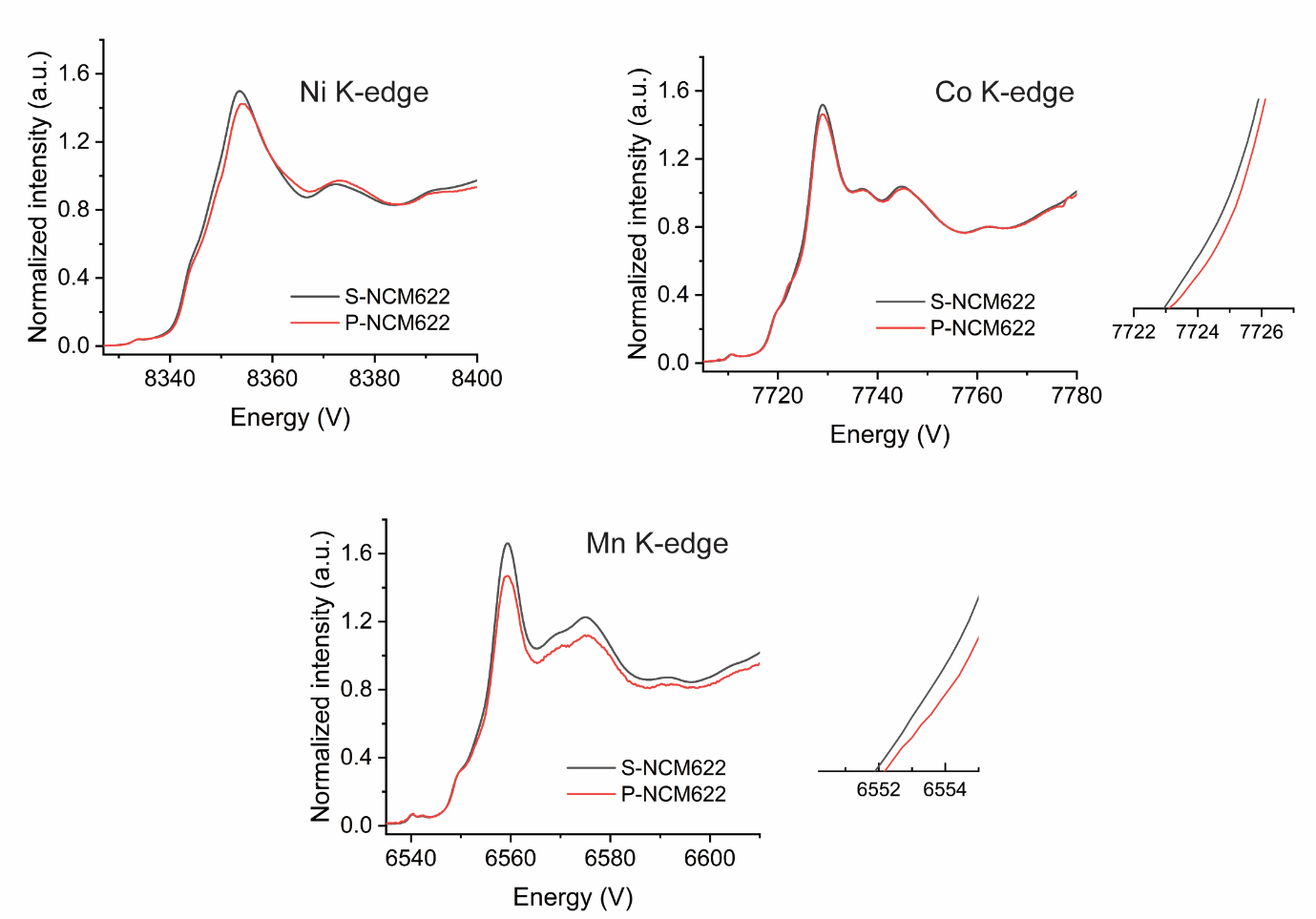


**Fig. S7.** Normalized XANES spectra of (c) Ni, (d) Co, and (e) Mn K-edge for P-NCM622 and S-NCM622 measured at ALBA.


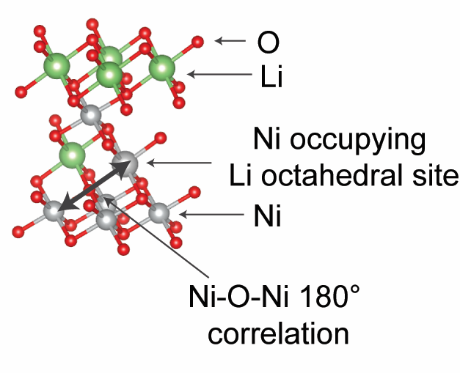


**Fig. S8.** Schematic illustration of Ni-O-Ni 180° correlation.


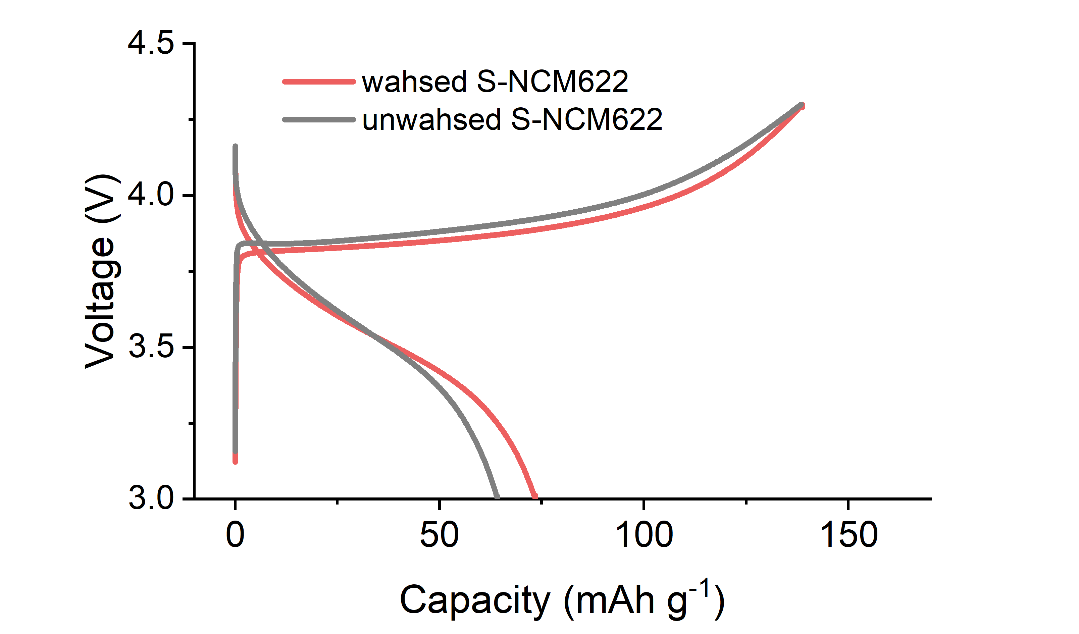


**Fig. S9.** Initial charge/discharge capacities at 0.1C over 3.0-4.3 V for S-NCM622 with and without water-washing process.


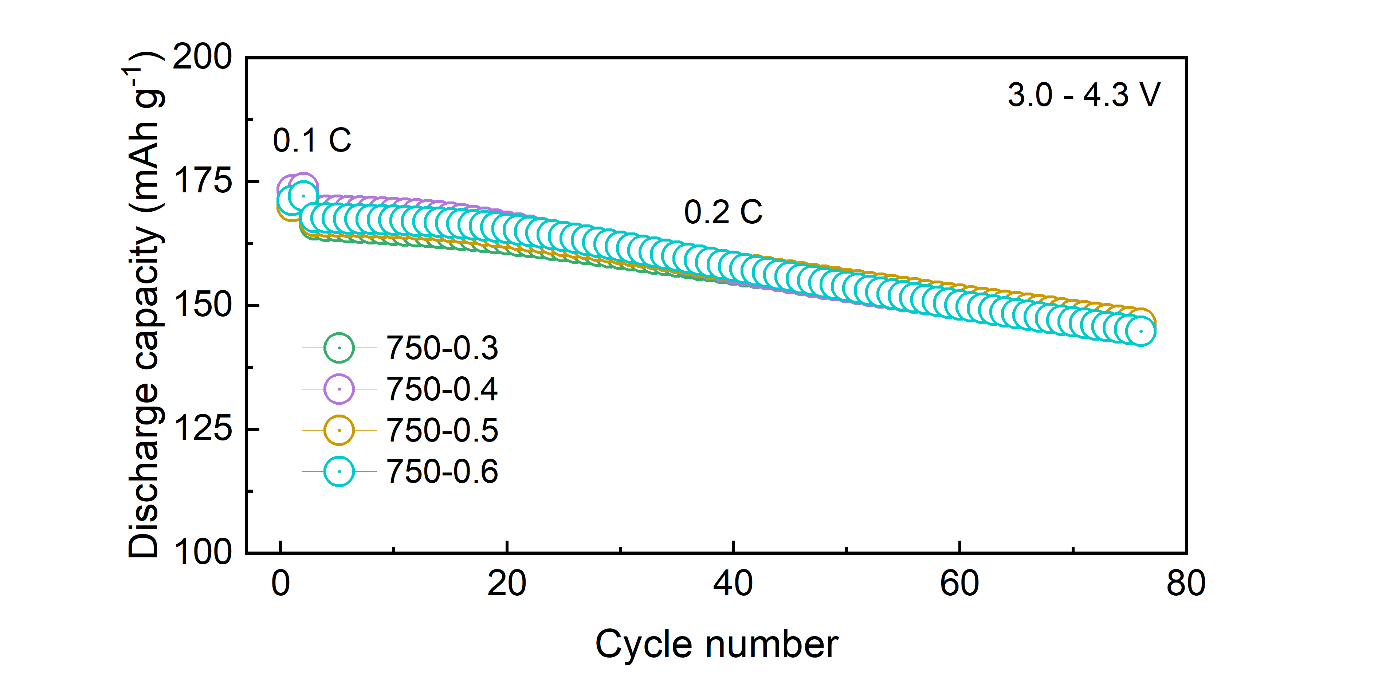


**Fig. S10.** Cycling performances of materials regenerated at 750°C with different Li/TM ratio.


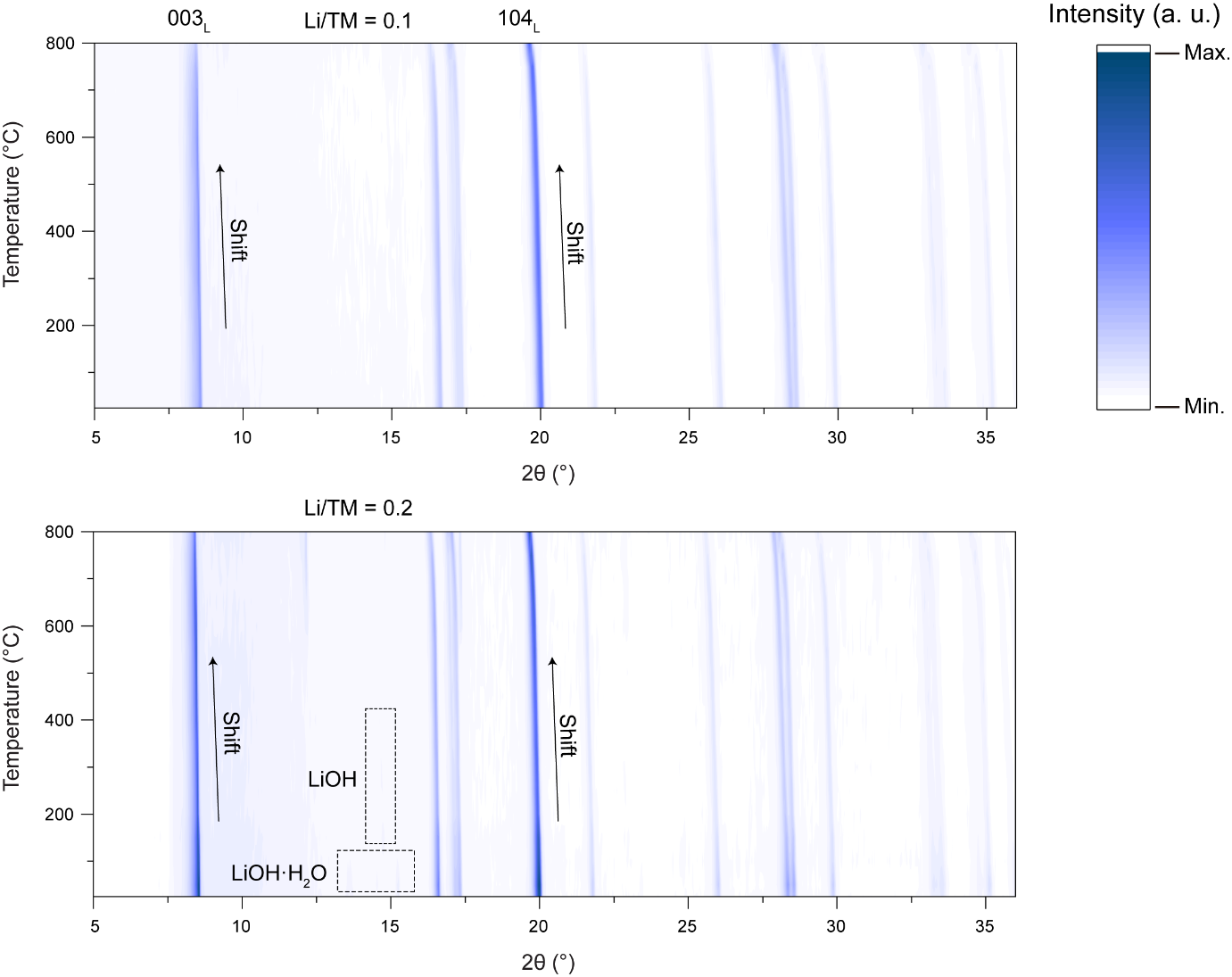


**Fig. S11.** In-situ high-temperature XRD patterns for Li/TM = 0.1 and Li/TM = 0.2.


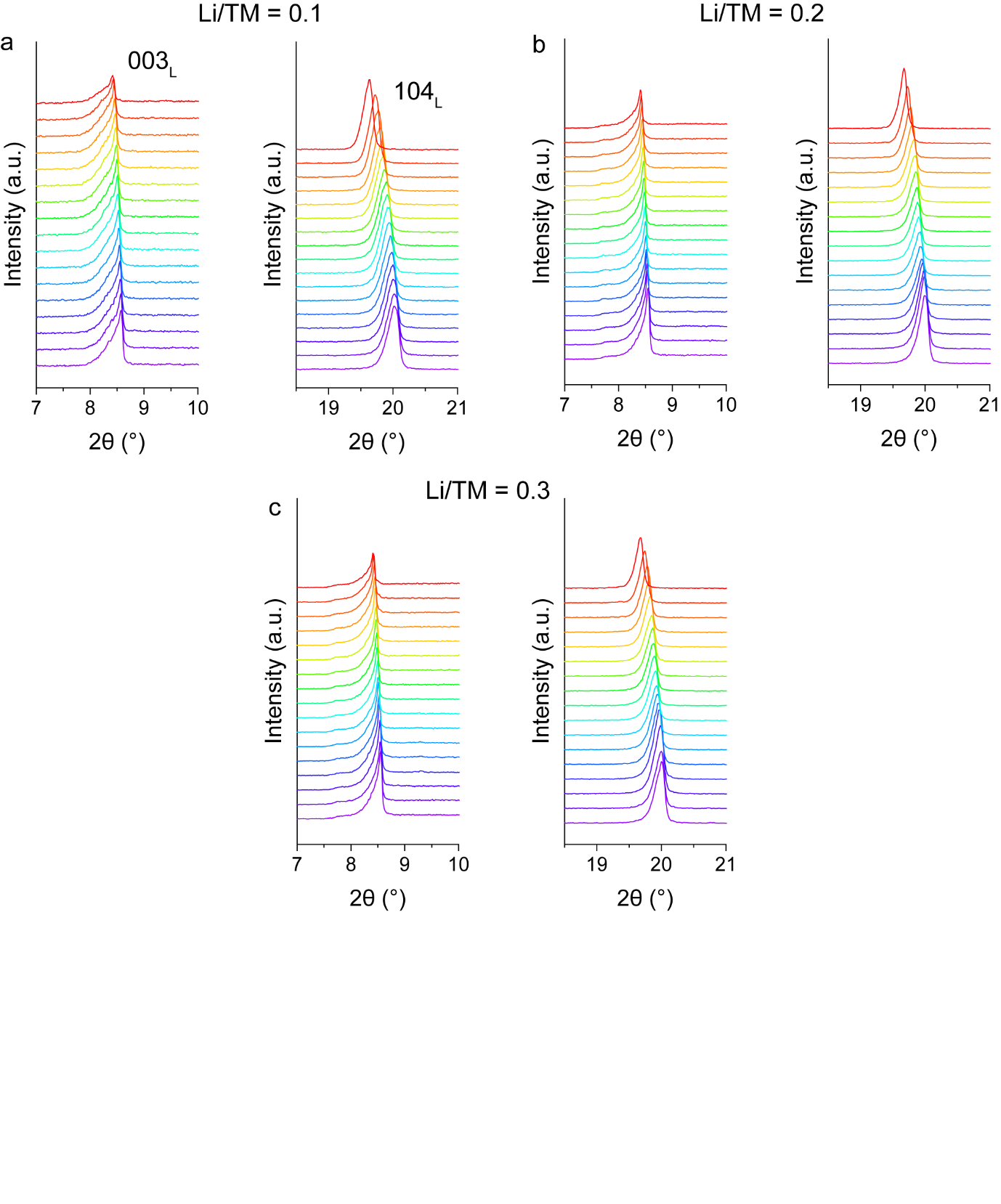


**Fig. S12.** Enlarged region of in-situ high-temperature XRD patterns for (a) Li/TM = 0.1, (b) Li/TM = 0.2 and (c) Li/TM = 0.3.


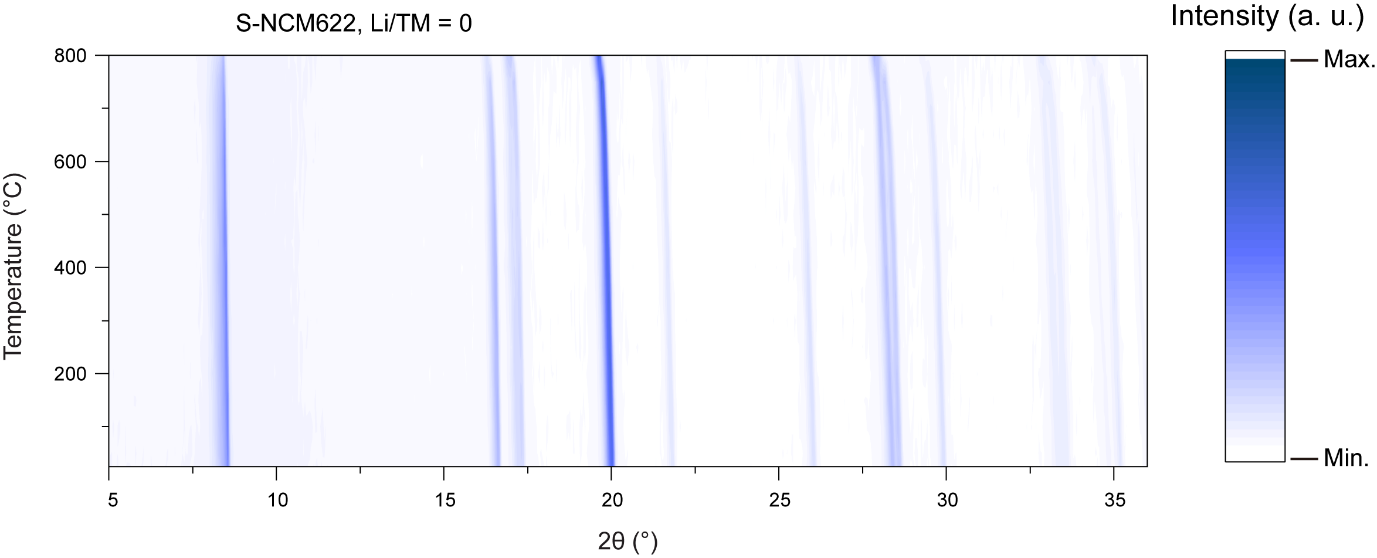


**Fig. S13.** In-situ high-temperature XRD patterns for S-NCM622.


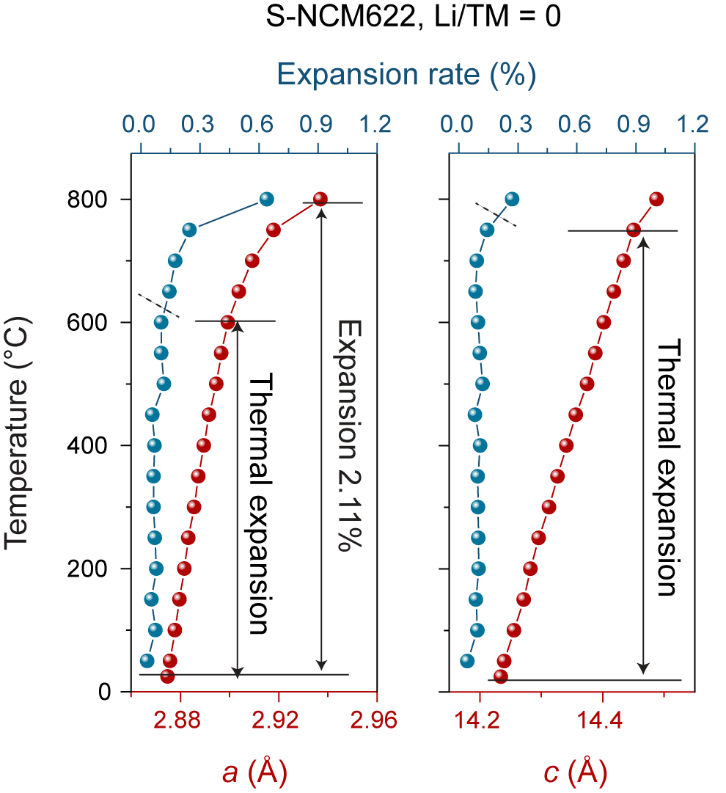


**Fig. S14.** Calculated *a* and *c* for S-NCM622.


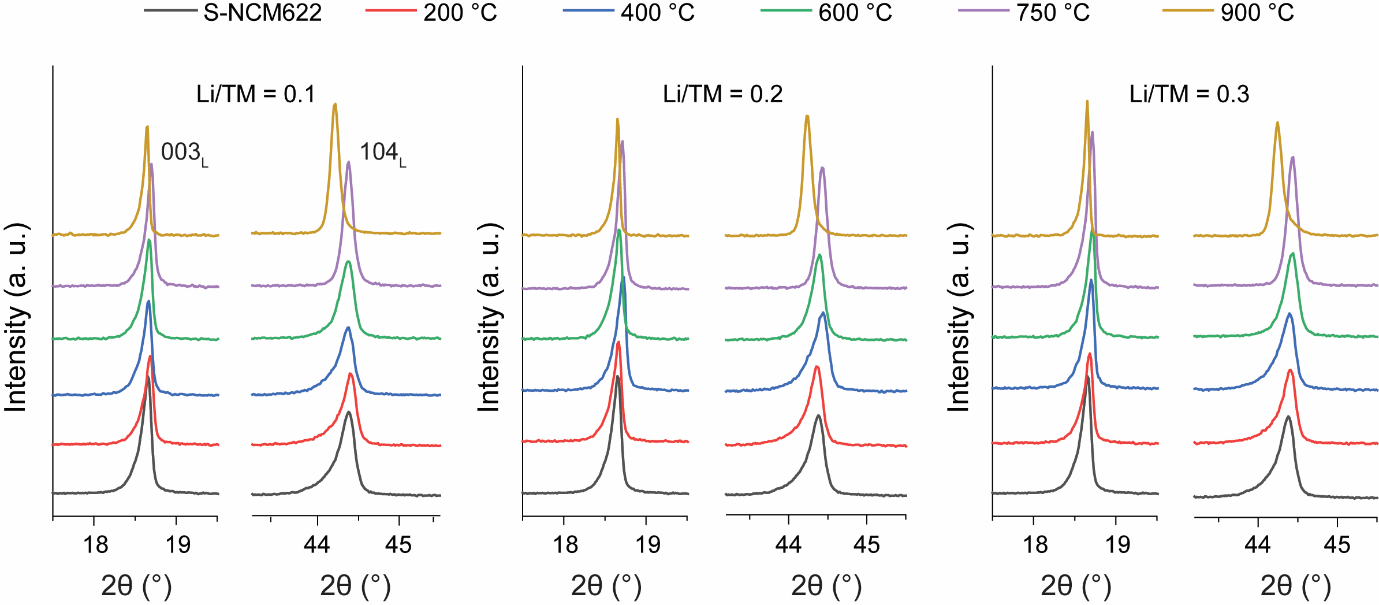


**Fig. S15.** Enlarged region of ex-situ XRD patterns for materials regenerated under different temperatures (200, 400 and 600°C) and Li/TM ratio of (0.1, 0.2 and 0.3).


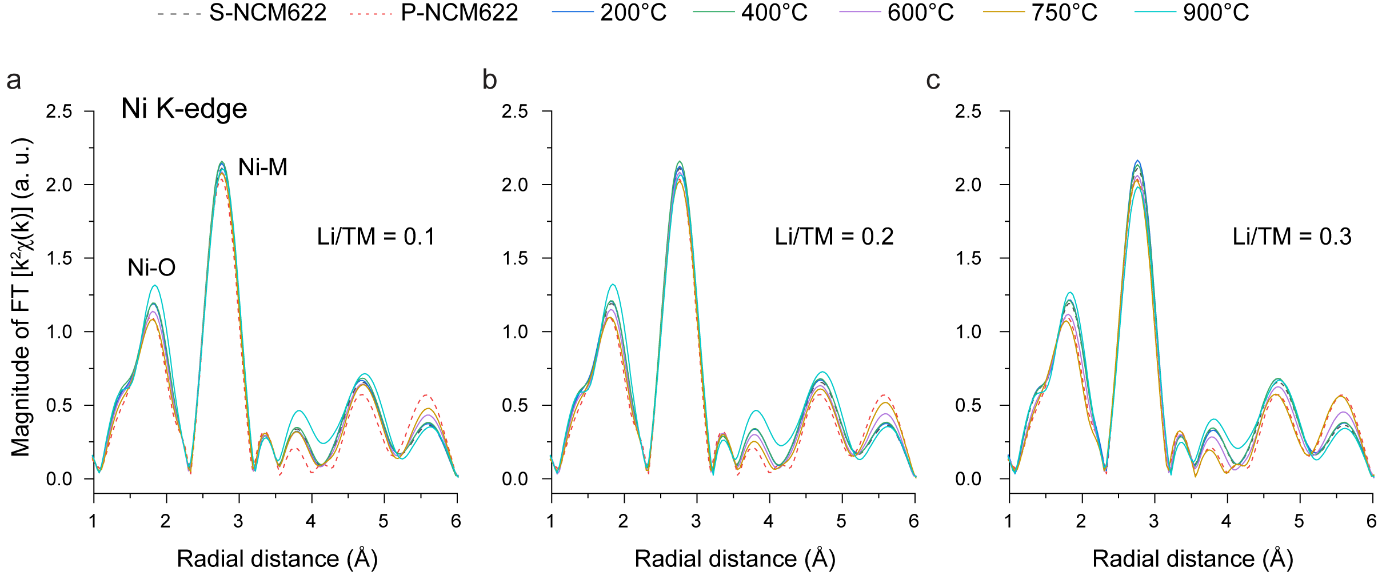


**Fig. S16.** EXAFS spectra of Ni K-edge for materials regenerated under different temperatures (200, 400, 600, 750 and 900°C) and Li/TM ratio of (0.1, 0.2 and 0.3).


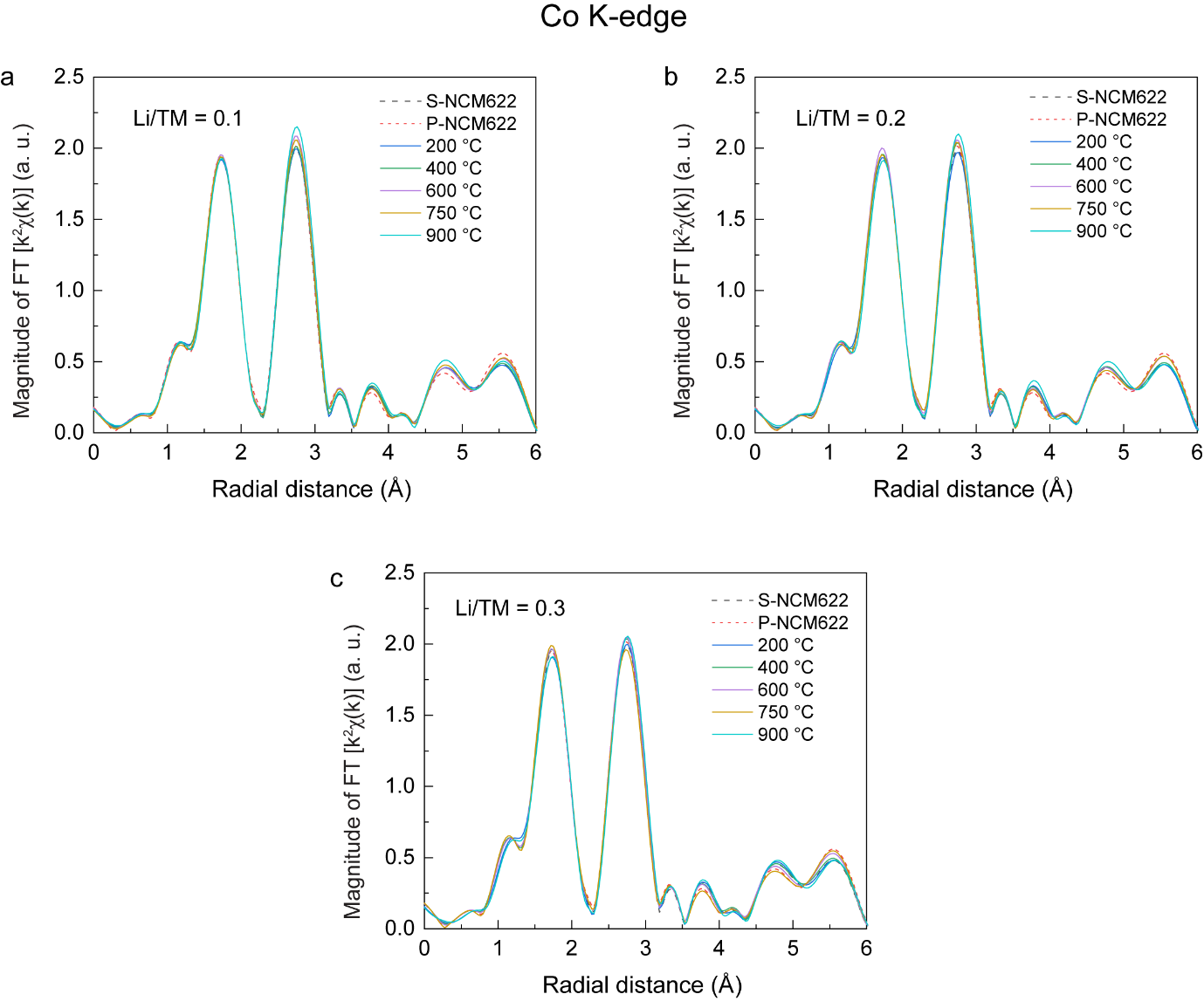


**Fig. S17.** EXAFS spectra of Co K-edge for materials regenerated under different temperatures (200, 400, 600, 750 and 900°C) with a Li/TM ratio of (a) 0.1, (b) 0.2 and (c) 0.3.


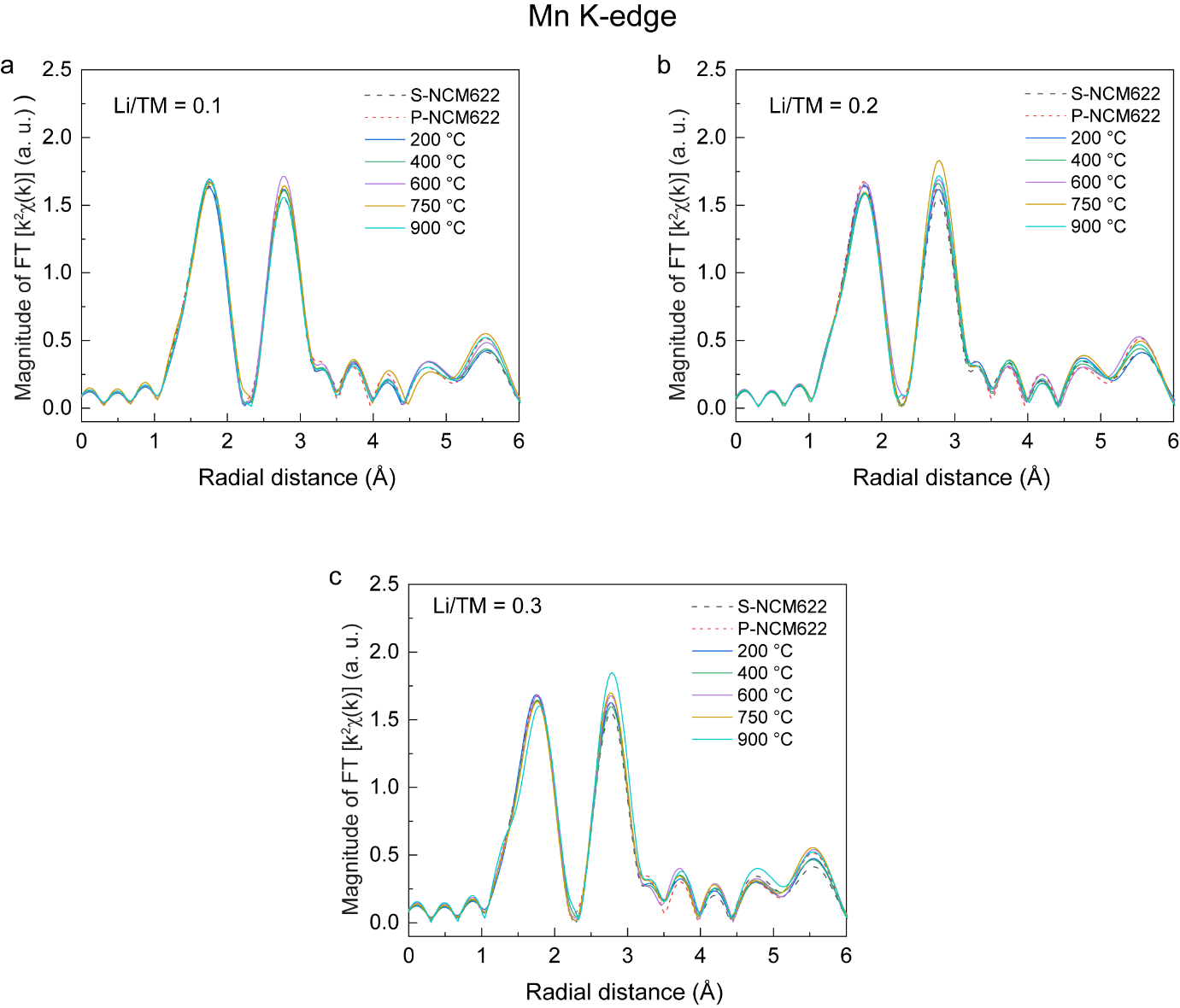


**Fig. S18.** EXAFS spectra of Mn K-edge for materials regenerated under different temperatures (200, 400, 600, 750 and 900°C) with a Li/TM ratio of (a) 0.1, (b) 0.2 and (c) 0.3.


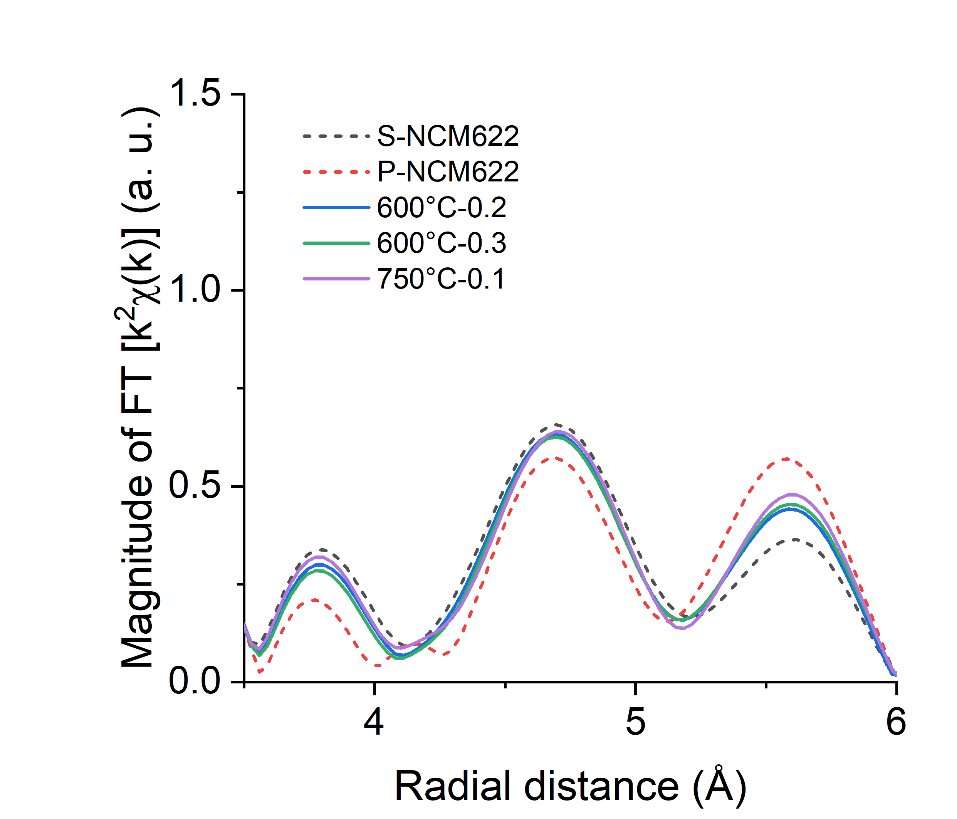


**Fig. S19.** Enlarged region of EXAFS spectra of Ni K-edge for materials regenerated at 600°C with the Li/TM ratios of 0.2 and 0.3 (600°C-0.2 and 600°C-0.3), and under 750 °C with the Li/TM ratios of 0.1 (750°C-0.1).


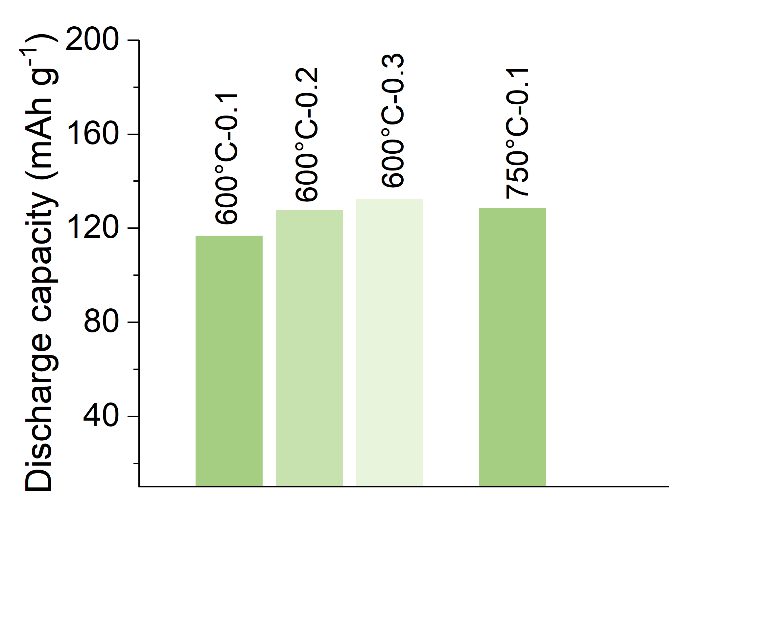


**Fig. S20.** Initial discharge capacities at 0.1C over 3.0-4.3 V for materials regenerated at 600°C with the Li/TM ratios of 0.1, 0.2 and 0.3 (600°C-0.1, 600°C-0.2 and 600°C-0.3), and under 750 °C with the Li/TM ratios of 0.1 (750°C-0.1).


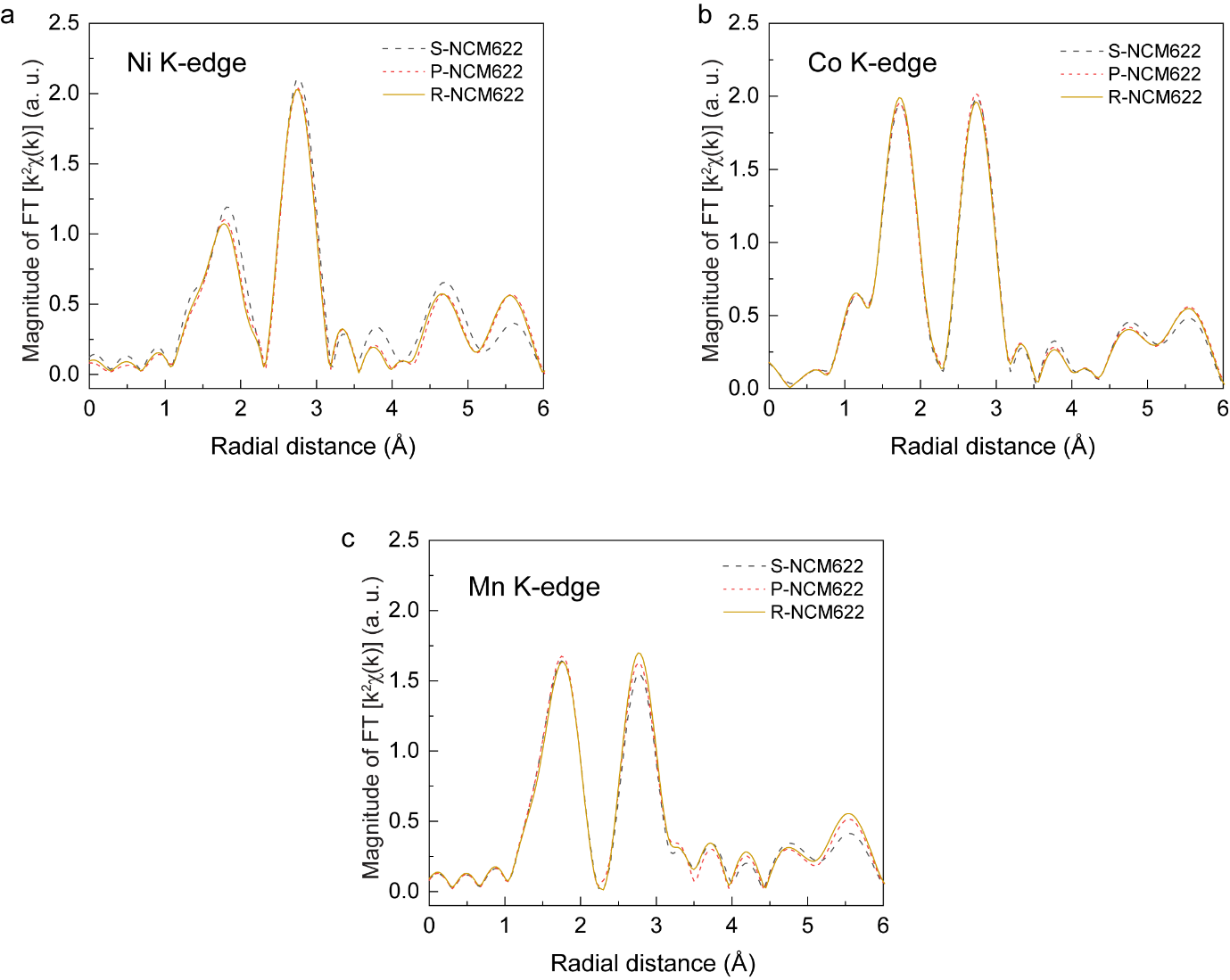


**Fig. S21.** EXAFS spectra of (a) Ni k-edge, (b) Co k-edge and (c) Mn k-edge for S-NCM622, P-NCM622 and R-NCM622.


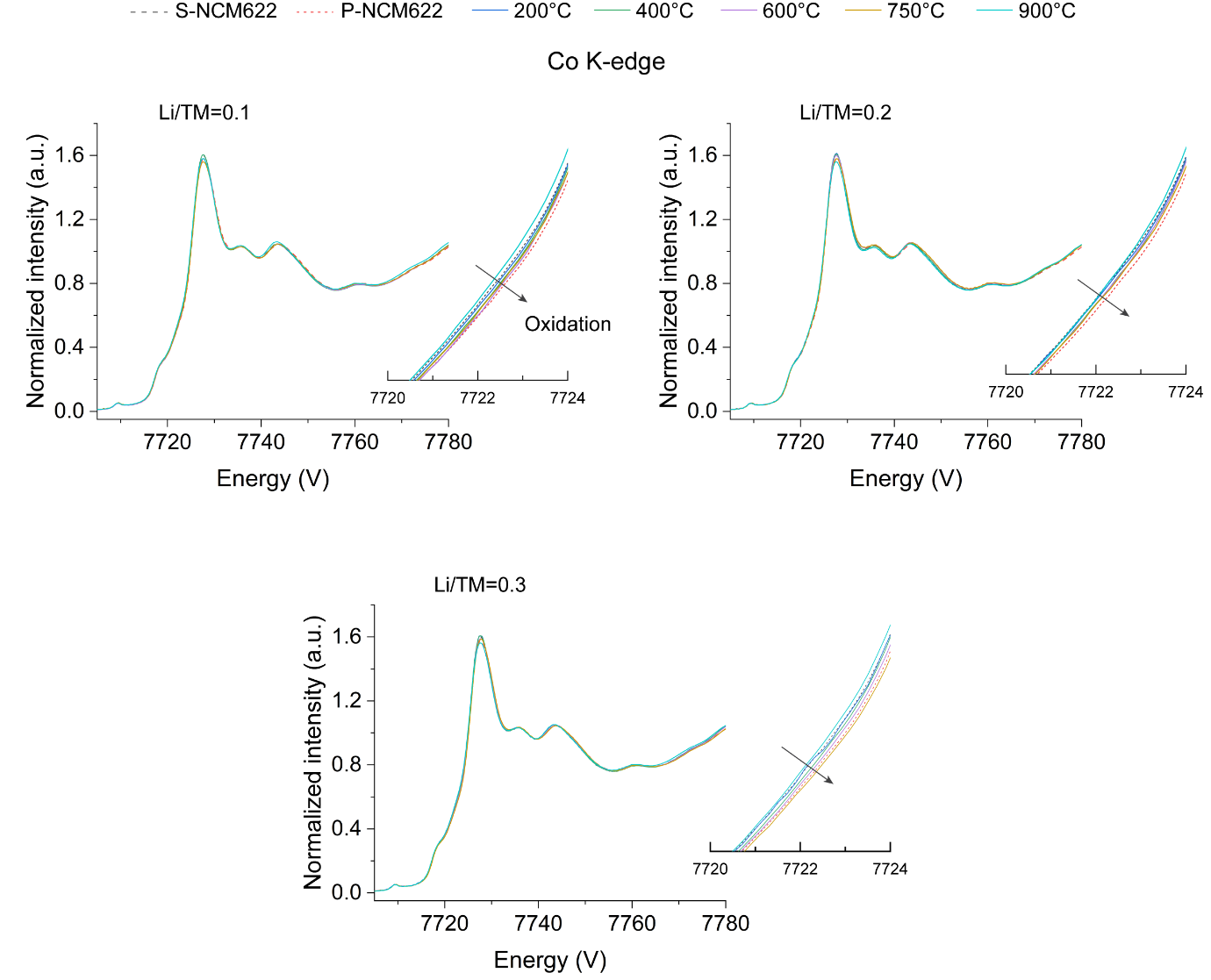


**Fig. S22.** Normalized XANES spectra of Co k-edge for materials regenerated under different temperatures (200, 400, 600, 750 and 900°C) with a Li/TM ratio of (d) 0.1, (e) 0.2 and (f) 0.3.


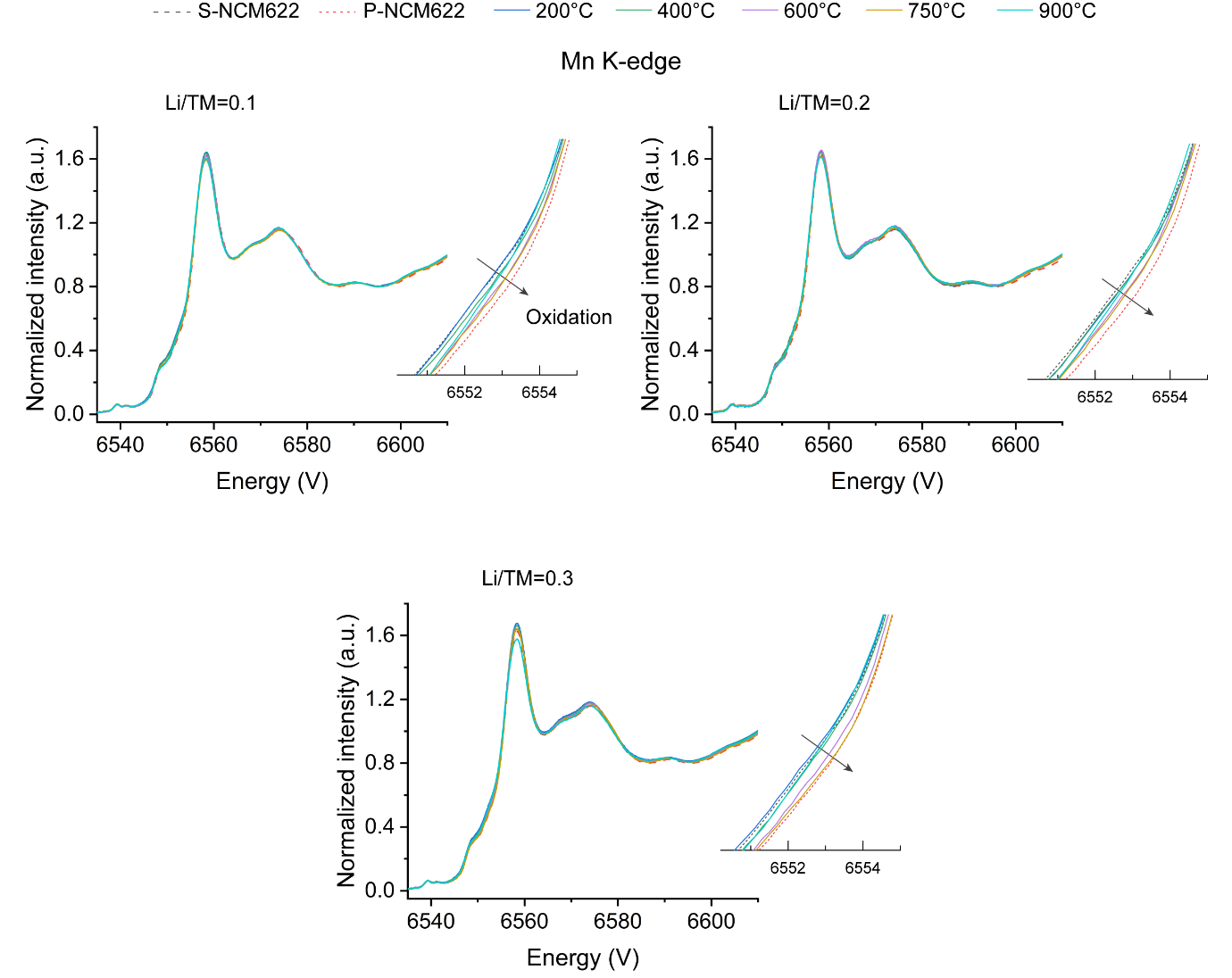


**Fig. S23.** Normalized XANES spectra of Mn k-edge for materials regenerated under different temperatures (200, 400, 600, 750 and 900°C) with a Li/TM ratio of (d) 0.1, (e) 0.2 and (f) 0.3.


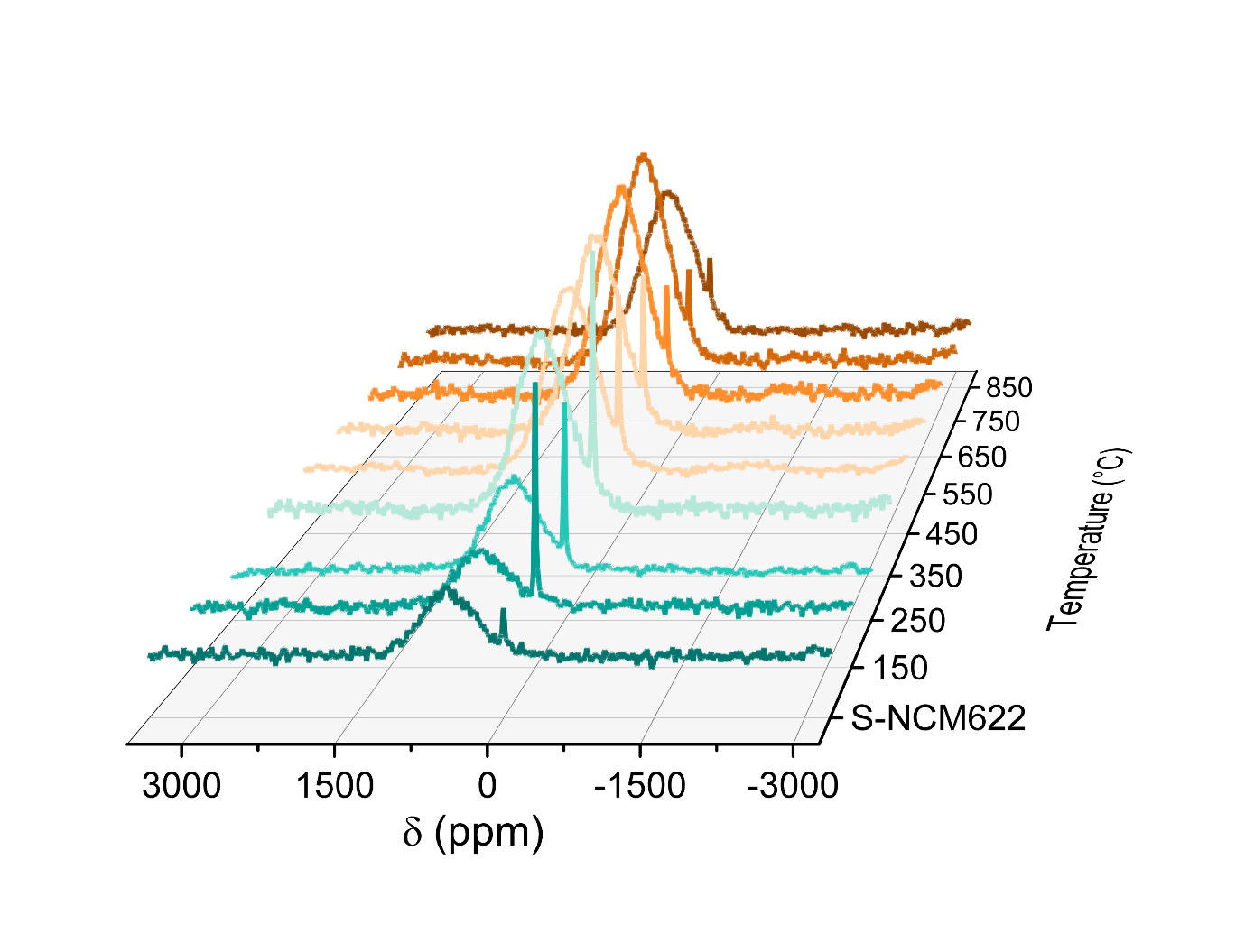


**Fig. S24.** ^6^Li solid-state NMR waterfall spectra for materials regenerated under different temperatures (150-850°C, per 100°C) with a Li/TM ratio of 0.3.


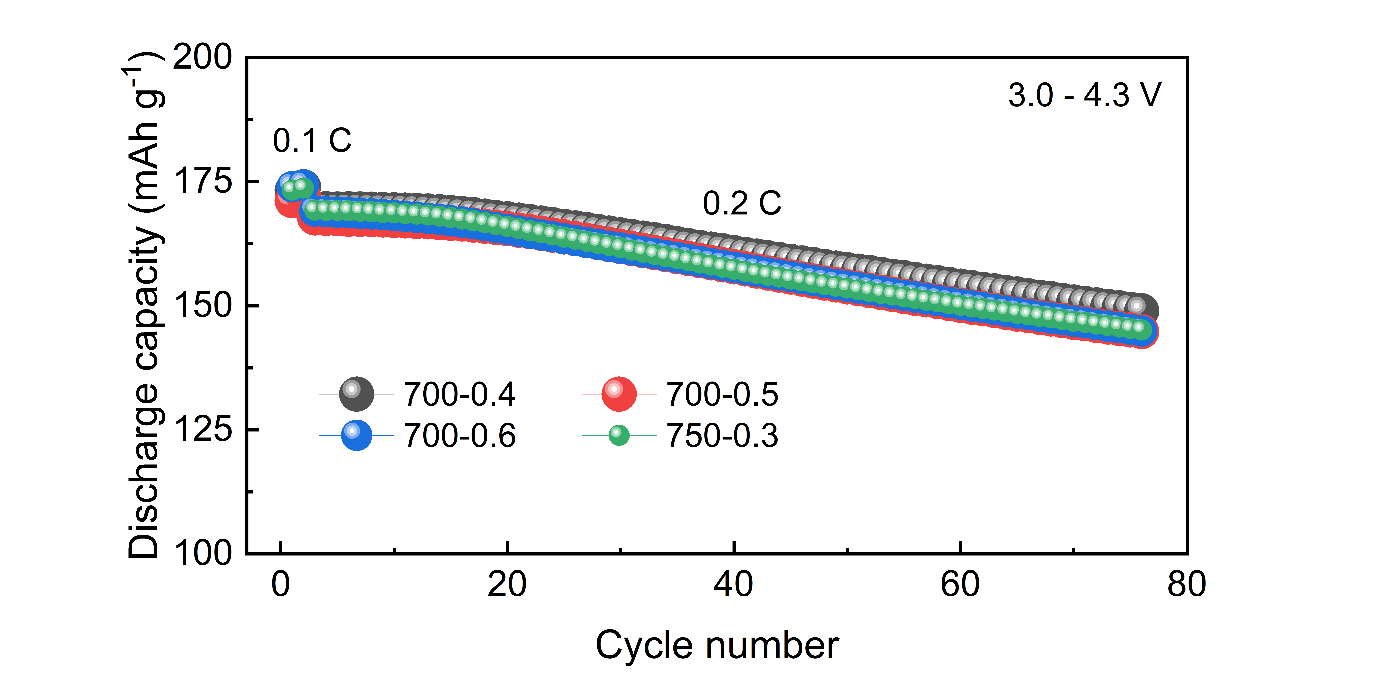


**Fig. S25.** Cycling performances of materials regenerated at 700 and 750°C with different Li/TM ratio.


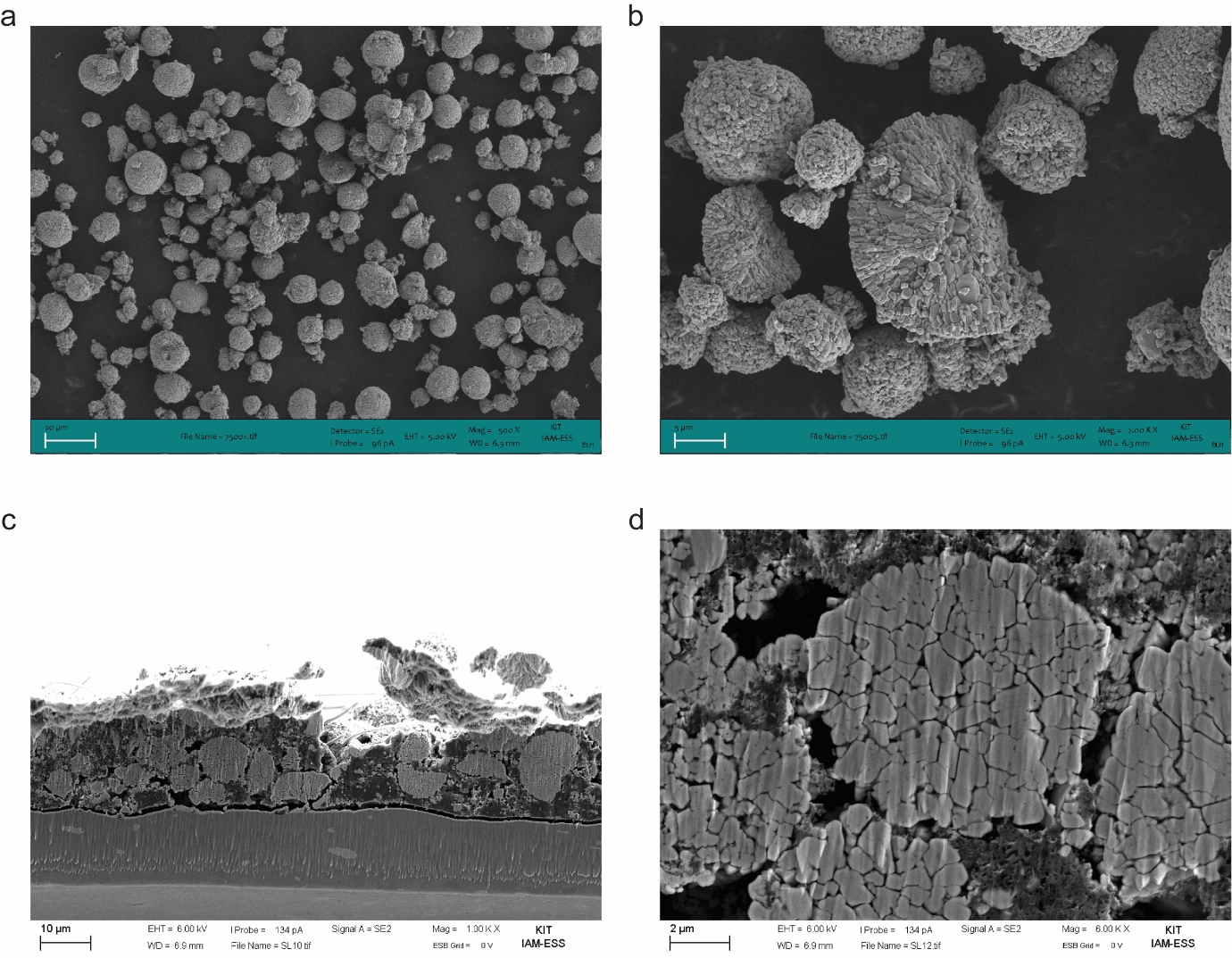


**Fig. S26.** SEM images of R-NCM622 (a-b) before and (c-d) after 200 cycles.


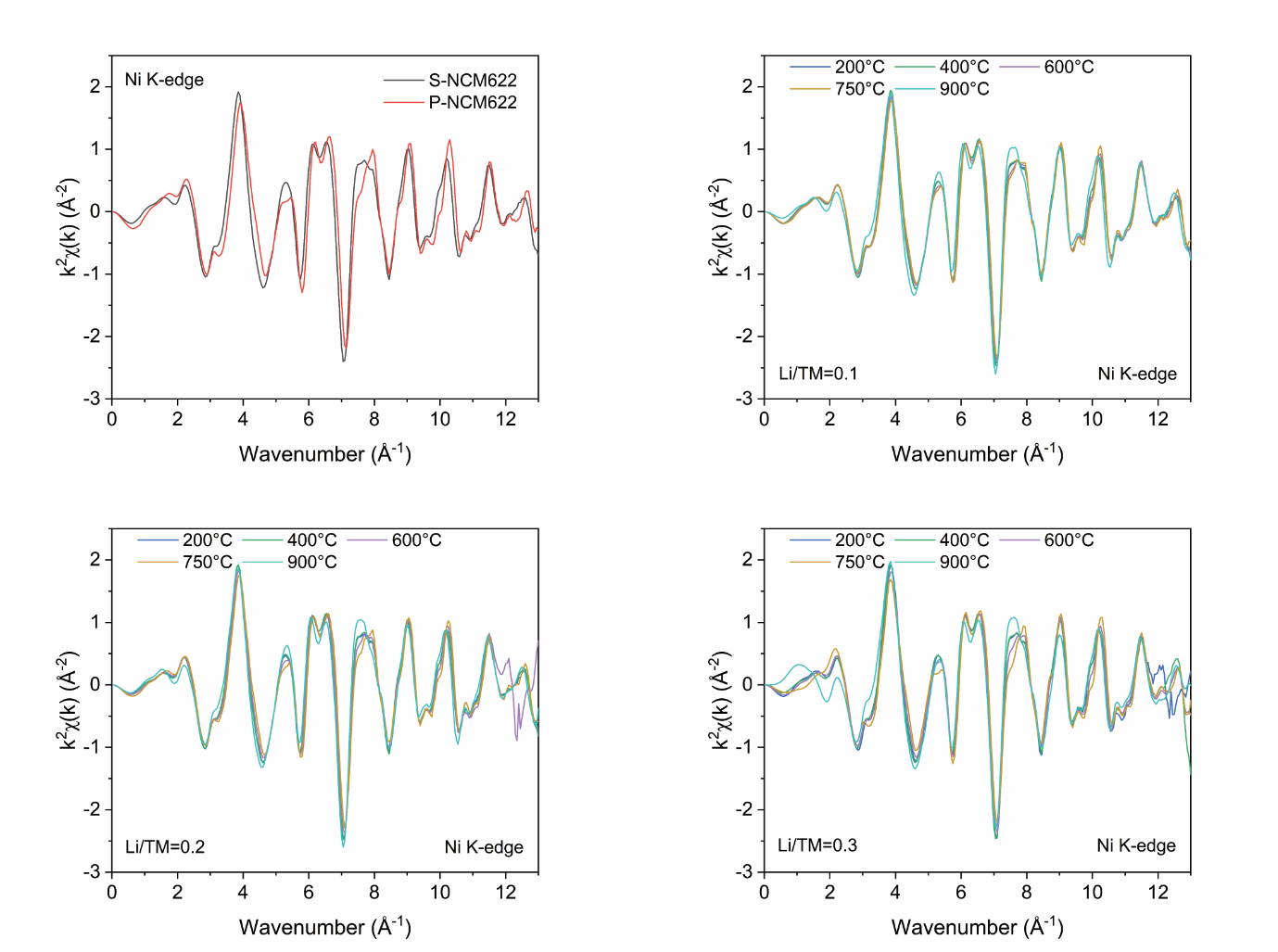


**Fig. S27.** Corresponding k^2^χ(k) data of Ni K-edge with a spline range from 0 to 13.0 Å^-1^.


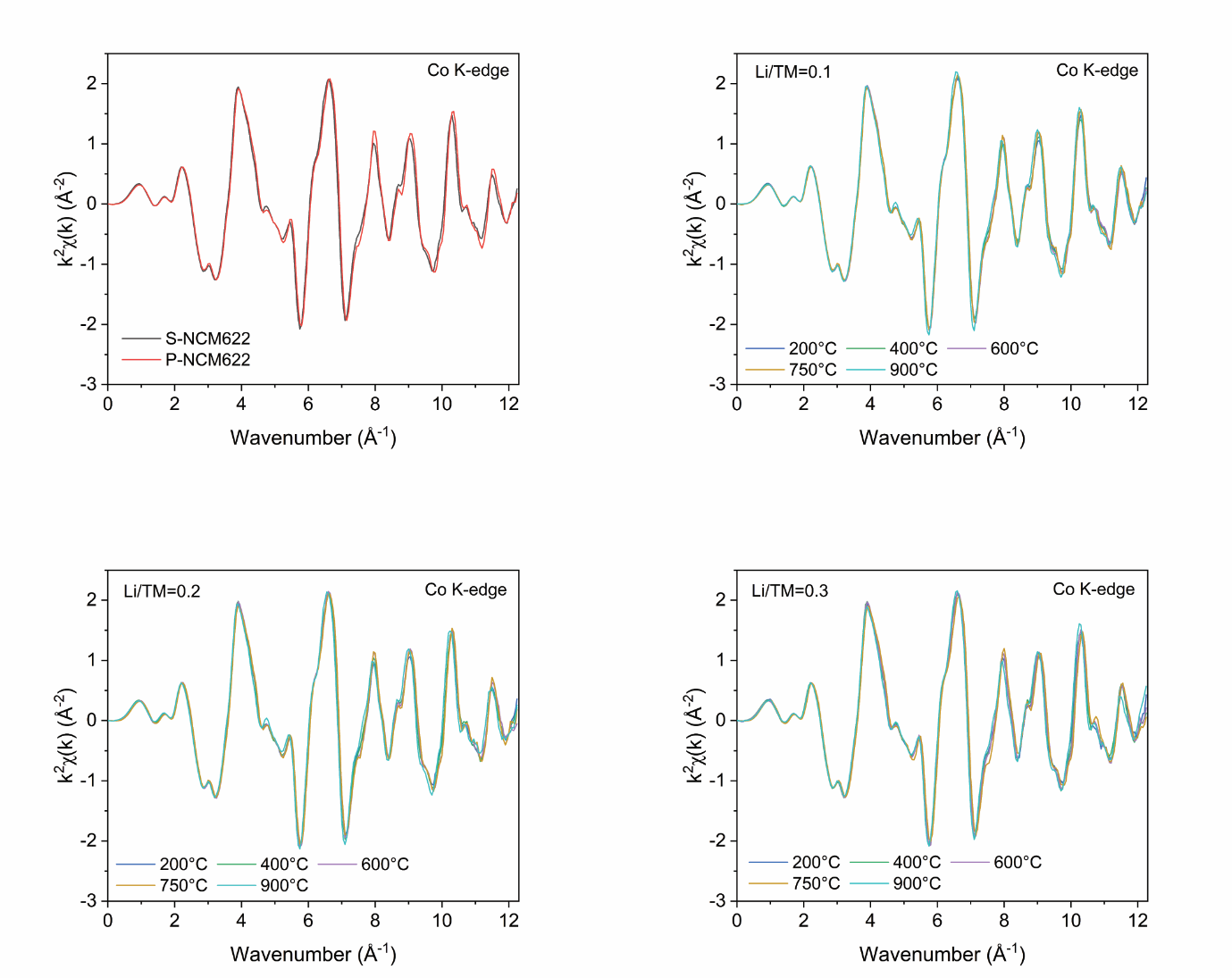


**Fig. S28.** Corresponding k^2^χ(k) data of Co K-edge with a spline range from 0 to 12.3 Å^-1^.


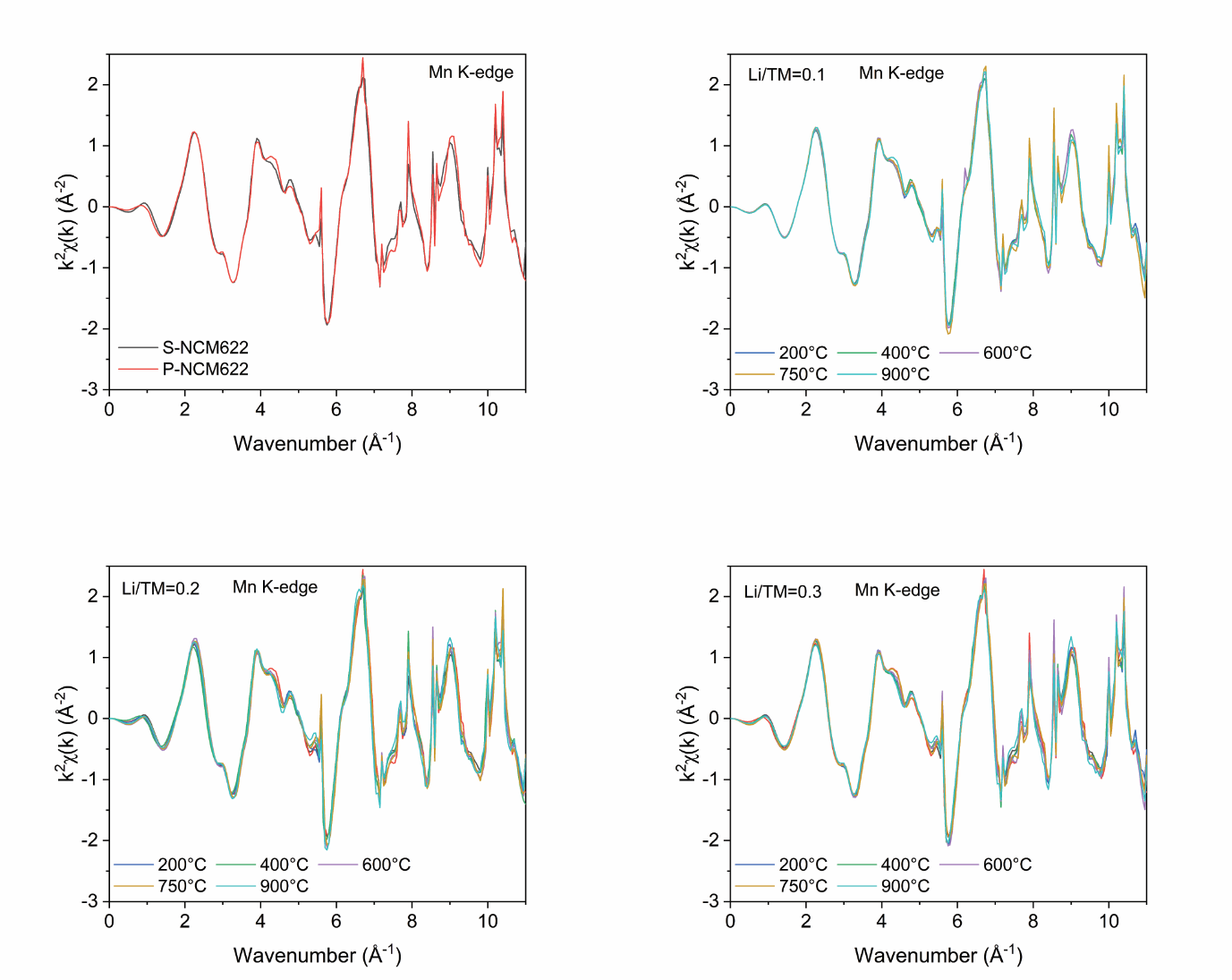


**Fig. S29.** Corresponding k^2^χ(k) data of Mn K-edge with a spline range from 0 to 11.0 Å^-1^.

**Tables**

**Table S1.** Elemental compositions from ICP-OES and TECG analyses for S-NCM622 without washing.

Avg: Average; SD: Standard deviation.

| *Weight percent (%)* | | | | | | | | | |
| --- | --- | --- | --- | --- | --- | --- | --- | --- | --- |
| Ni | | Co | | Mn | | Li | | O | |
| Avg | SD | Avg | SD | Avg | SD | Avg | SD | Avg | SD |
| 35.6 | 0.7 | 11.8 | 0.3 | 11.1 | 0.2 | 6.83 | 0.12 | 31.0 | 1.0 |

**Table S2.** Elemental compositions from ICP-OES and TECG analyses for S-NCM622 with washing.

Avg: Average; SD: Standard deviation.

| *Weight percent (%)* | | | | | | | | | |
| --- | --- | --- | --- | --- | --- | --- | --- | --- | --- |
| Ni | | Co | | Mn | | Li | | O | |
| Avg | SD | Avg | SD | Avg | SD | Avg | SD | Avg | SD |
| 36.8 | 0.3 | 12.2 | 0.1 | 11.5 | 0.1 | 6.02 | 0.06 | 31.8 | 0.7 |

**Table S3.** Elemental compositions from ICP-OES and TECG analyses for P-NCM622.

Avg: Average; SD: Standard deviation.

| *Weight percent (%)* | | | | | | | | | |
| --- | --- | --- | --- | --- | --- | --- | --- | --- | --- |
| Ni | | Co | | Mn | | Li | | O | |
| Avg | SD | Avg | SD | Avg | SD | Avg | SD | Avg | SD |
| 34.5 | 0.5 | 11.4 | 0.2 | 10.7 | 0.1 | 6.88 | 0.4 | 31.8 | 0.4 |

**Table S4.** Cell parameters from in-situ high-temperature XRD of Li/TM=0.1.

| Temperature (℃) | *a* (*b)* (Å) | *c* (Å) | Volume (Å^3^) | *R_p_, %* | *R_wp_, %* |
| --- | --- | --- | --- | --- | --- |
| 25 | 2.87488(11) | 14.23453(95) | 101.886(9) | 9.35 | 11.8 |
| 50 | 2.87578(12) | 14.23970(102) | 101.987(9) | 10.1 | 12.5 |
| 100 | 2.87782(11) | 14.25595(93) | 102.248(9) | 9.23 | 11.7 |
| 150 | 2.87966(11) | 14.27182(93) | 102.493(9) | 9.22 | 11.8 |
| 200 | 2.88168(12) | 14.28239(103) | 102.713(9) | 10.4 | 12.9 |
| 250 | 2.88325 (11) | 14.29603(93) | 102.922(9) | 8.12 | 12.60 |
| 300 | 2.88560(11) | 14.31286(95) | 103.211(9) | 9.50 | 12.1 |
| 350 | 2.88731(11) | 14.32664(95) | 103.433(9) | 9.68 | 12.3 |
| 400 | 2.88964(11) | 14.34110(96) | 103.705(9) | 10.1 | 12.6 |
| 450 | 2.89171(11) | 14.35646(96) | 103.965(9) | 10.0 | 12.4 |
| 500 | 2.89466(11) | 14.37479(99) | 104.310(9) | 10.5 | 13.0 |
| 550 | 2.89667(11) | 14.38811(94) | 104.552(9) | 9.89 | 12.6 |
| 600 | 2.89931(10) | 14.40188(89) | 104.843(8) | 9.41 | 11.9 |
| 650 | 2.90386(10) | 14.41852(90) | 105.294(8) | 9.69 | 12.1 |
| 700 | 2.90922(10) | 14.43439(87) | 105.799(8) | 9.67 | 12.3 |
| 750 | 2.91798 (10) | 14.45082(77) | 106.558(8) | 7.85 | 9.96 |
| 800 | 2.93703(11) | 14.48818(113) | 108.233(11) | 9.51 | 12.1 |

**Table S5.** Cell parameters from in-situ high-temperature XRD of Li/TM=0.2.

| Temperature (℃) | *a* (*b)* (Å) | *c* (Å) | Volume (Å^3^) | *R_p_, %* | *R_wp_, %* |
| --- | --- | --- | --- | --- | --- |
| 25 | 2.87948(8) | 14.25732(69) | 102.378(6) | 8.52 | 11.7 |
| 50 | 2.87949(8) | 14.25815(68) | 102.382(6) | 8.91 | 12.4 |
| 100 | 2.88198(8) | 14.27453(68) | 102.675(6) | 8.35 | 11.3 |
| 150 | 2.88286(8) | 14.28423(96) | 102.810(6) | 8.32 | 11.2 |
| 200 | 2.88546(8) | 14.30105(74) | 103.117(7) | 8.34 | 11.3 |
| 250 | 2.8875(8) | 14.31794(71) | 103.384(7) | 7.95 | 10.7 |
| 300 | 2.88947(8) | 14.32959(74) | 103.609(7) | 8.07 | 11.0 |
| 350 | 2.89125(8) | 14.34422(70) | 103.844(6) | 7.94 | 10.7 |
| 400 | 2.89288(8) | 14.35818(71) | 104.062(7) | 7.93 | 10.6 |
| 450 | 2.89483(8) | 14.37132(72) | 104.297(7) | 8.22 | 11.1 |
| 500 | 2.89677(7) | 14.38566(68) | 104.541(6) | 7.60 | 10.1 |
| 550 | 2.89855(7) | 14.39890(65) | 104.766(6) | 7.54 | 10.0 |
| 600 | 2.90112(8) | 14.41441(75) | 105.065(7) | 8.36 | 11.6 |
| 650 | 2.90370(9) | 14.42750(86) | 105.348(8) | 9.57 | 13.3 |
| 700 | 2.90919(9) | 14.44566(81) | 105.879(8) | 9.29 | 14.2 |
| 750 | 2.91572(10) | 14.46214(82) | 106.558(8) | 9.05 | 14.7 |
| 800 | 2.92637(10) | 14.48210(79) | 107.404(8) | 8.80 | 13.6 |

**Table S6.** Cell parameters from in-situ high-temperature XRD of Li/TM=0.3.

| Temperature (℃) | *a* (*b)* (Å) | *c* (Å) | Volume (Å^3^) | *R_p_, %* | *R_wp_, %* |
| --- | --- | --- | --- | --- | --- |
| 25 | 2.87949(9) | 14.26081(80) | 102.401(7) | 8.89 | 12.4 |
| 50 | 2.88006(9) | 14.26360(78) | 102.462(7) | 8.68 | 12.1 |
| 100 | 2.88075(8) | 14.27195(71) | 102.571(6) | 8.87 | 12.2 |
| 150 | 2.88258(8) | 14.28289(70) | 102.780(7) | 9.03 | 12.4 |
| 200 | 2.88468(8) | 14.29644(70) | 103.027(6) | 8.83 | 11.3 |
| 250 | 2.88687(8) | 14.31069(72) | 103.287(7) | 8.60 | 11.7 |
| 300 | 2.88847(8) | 14.32522(72) | 103.506(7) | 8.14 | 11.0 |
| 350 | 2.89068(8) | 14.34158(69) | 103.783(6) | 7.78 | 10.6 |
| 400 | 2.89228(8) | 14.35467(68) | 103.993(6) | 7.69 | 10.4 |
| 450 | 2.89403(8) | 14.36713(71) | 104.209(7) | 7.91 | 10.7 |
| 500 | 2.89605(8) | 14.38006(73) | 104.449(7) | 8.04 | 10.8 |
| 550 | 2.8979(8) | 14.39579(70) | 104.697(6) | 7.80 | 10.4 |
| 600 | 2.90052(9) | 14.41186(72) | 105.003(7) | 8.26 | 11.6 |
| 650 | 2.90265(9) | 14.42301(76) | 105.239(7) | 8.19 | 12.0 |
| 700 | 2.90631(9) | 14.43962(83) | 105.626(8) | 8.68 | 13.4 |
| 750 | 2.91394(13) | 14.46186(107) | 106.345(10) | 11.5 | 18.1 |
| 800 | 2.92665(17) | 14.48510(132) | 107.447(13) | 12.0 | 19.8 |

**Table S7.** Cell parameters from in-situ high-temperature XRD of P-NCM622.

| Temperature (℃) | *a* (*b)* (Å) | *c* (Å) | Volume (Å^3^) | *R_p_, %* | *R_wp_, %* |
| --- | --- | --- | --- | --- | --- |
| 25 | 2.86673(8) | 14.21325(65) | 101.157(6) | 9.87 | 12.3 |
| 50 | 2.86735(7) | 14.21967(64) | 101.247(6) | 9.65 | 12.0 |
| 100 | 2.86891(8) | 14.23361(70) | 101.456(6) | 10.5 | 13.3 |
| 150 | 2.87068(7) | 14.24519(65) | 101.665(6) | 9.73 | 12.3 |
| 200 | 2.87259(8) | 14.25927(68) | 101.901(6) | 10.3 | 12.9 |
| 250 | 2.87407(7) | 14.27140(63) | 102.092(6) | 9.34 | 12.1 |
| 300 | 2.87640(8) | 14.28693(65) | 102.369(6) | 10.0 | 12.6 |
| 350 | 2.87842(7) | 14.30260(64) | 102.625(6) | 9.95 | 12.6 |
| 400 | 2.88066(8) | 14.31755(66) | 102.893(6) | 10.3 | 12.9 |
| 450 | 2.88273(7) | 14.33168(65) | 103.142(6) | 9.76 | 12.5 |
| 500 | 2.88492(8) | 14.34836(68) | 103.419(6) | 10.3 | 13.0 |
| 550 | 2.88713(7) | 14.36418(65) | 103.692(6) | 9.65 | 12.5 |
| 600 | 2.89001(8) | 14.38258(68) | 104.032(6) | 9.76 | 12.6 |
| 650 | 2.89266(8) | 14.39924(67) | 104.343(6) | 9.93 | 12.5 |
| 700 | 2.89533(8) | 14.41372(68) | 104.641(6) | 9.86 | 12.5 |
| 750 | 2.89900(8) | 14.42951(67) | 105.022(6) | 9.72 | 12.2 |
| 800 | 2.90317(8) | 14.44634(65) | 105.447(8) | 8.95 | 11.8 |

**Table S8.** Cell parameters from in-situ high-temperature XRD of S-NCM622.

| Temperature (℃) | *a* (*b)* (Å) | *c* (Å) | Volume (Å^3^) | *R_p_, %* | *R_wp_, %* |
| --- | --- | --- | --- | --- | --- |
| 25 | 2.87586(10) | 14.24250(90) | 102.012(8) | 8.66 | 11.1 |
| 50 | 2.87676(11) | 14.24870(97) | 102.120(9) | 9.71 | 12.2 |
| 100 | 2.87890(11) | 14.26205(96) | 102.368(9) | 9.52 | 12.0 |
| 150 | 2.88045(10) | 14.27441(91) | 102.568(8) | 9.12 | 11.6 |
| 200 | 2.88271(11) | 14.28873(95) | 102.832(9) | 9.64 | 12.2 |
| 250 | 2.88475(11) | 14.30290(99) | 103.079(9) | 10.1 | 12.6 |
| 300 | 2.88659(11) | 14.31684(101) | 103.311(9) | 10.6 | 13.0 |
| 350 | 2.88844(11) | 14.33045(96) | 103.796(9) | 10.2 | 12.6 |
| 400 | 2.89042(10) | 14.34598(92) | 103.705(9) | 9.30 | 12.0 |
| 450 | 2.89212(11) | 14.35782(89) | 104.004(8) | 9.24 | 11.6 |
| 500 | 2.89551(11) | 14.37507(93) | 104.374(9) | 9.66 | 11.9 |
| 550 | 2.89848(10) | 14.39045(85) | 104.700(8) | 8.52 | 10.7 |
| 600 | 2.90148(10) | 14.40456(86) | 105.019(8) | 8.49 | 10.8 |
| 650 | 2.90568(9) | 14.41688(79) | 105.414(8) | 8.06 | 10.1 |
| 700 | 2.91074(10) | 14.42997(81) | 105.877(8) | 8.44 | 10.4 |
| 750 | 2.91794(9) | 14.45075(76) | 106.555(7) | 7.60 | 9.73 |
| 800 | 2.93662(12) | 14.48990(103) | 108.233(10) | 8.42 | 10.9 |

**Table S9.** Elemental compositions from ICP-OES and TECG analyses for material regenerated at 750℃ with a Li/TM ratio of 0.3.

Avg: Average; SD: Standard deviation.

| *Weight percent (%)* | | | | | | | | | |
| --- | --- | --- | --- | --- | --- | --- | --- | --- | --- |
| Ni | | Co | | Mn | | Li | | O | |
| Avg | SD | Avg | SD | Avg | SD | Avg | SD | Avg | SD |
| 35.8 | 0.1 | 12.0 | 0.1 | 11.2 | 0.1 | 6.88 | 0.10 | 35.8 | 0.1 |

**Table S10.** Elemental compositions from ICP-OES and TECG analyses regenerated at 800℃ with a Li/TM ratio of 0.3.

Avg: Average; SD: Standard deviation.

| *Weight percent (%)* | | | | | | | | | |
| --- | --- | --- | --- | --- | --- | --- | --- | --- | --- |
| Ni | | Co | | Mn | | Li | | O | |
| Avg | SD | Avg | SD | Avg | SD | Avg | SD | Avg | SD |
| 36.0 | 0.1 | 12.1 | 0.1 | 11.4 | 0.1 | 6.78 | 0.10 | 32.6 | 0.2 |

**References**

(1) Smith, A.; Stüble, P.; Leuthner, L.; Hofmann, A.; Jeschull, F.; Mereacre, L. Potential and Limitations of Research Battery Cell Types for Electrochemical Data Acquisition. *Batteries & Supercaps* **2023**, *6* (6), e202300080.

(2) Liu, S.; Dolotko, O.; Bergfeldt, T.; Ehrenberg, H.; Knapp, M. Towards Sustainable Direct Recycling: Unraveling Structural Degradation Induced by Thermal Pretreatment of Lithium-Ion Battery Electrodes. *ChemSusChem* **2025**, *18* (1), e202400727.

(3) Rodríguez-Carvajal, J. FullProf. *CEA/Saclay, France* **2001**, *1045*, 132-146.

(4) Ravel, B.; Newville, M. ATHENA, ARTEMIS, HEPHAESTUS: data analysis for X-ray absorption spectroscopy using IFEFFIT. *Journal of synchrotron radiation* **2005**, *12* (4), 537-541.

(5) Li, H.; Hua, W.; Liu-Théato, X.; Fu, Q.; Desmau, M.; Missyul, A.; Knapp, M.; Ehrenberg, H.; Indris, S. New Insights into Lithium Hopping and Ordering in LiNiO_2_ Cathodes during Li (De)intercalation. *Chemistry of Materials* **2021**, *33* (24), 9546-9559.

(6) Bielecki, A.; Burum, D. P. Temperature dependence of ^207^Pb MAS spectra of solid lead nitrate. an accurate, sensitive thermometer for variable-temperature MAS. *Journal of Magnetic Resonance, Series A* **1995**, *116* (2), 215-220.
